# Population Histories and Genomic Diversity of South American Natives

**DOI:** 10.1093/molbev/msab339

**Published:** 2021-12-08

**Authors:** Marcos Araújo Castro e Silva, Tiago Ferraz, Cainã M Couto-Silva, Renan B Lemes, Kelly Nunes, David Comas, Tábita Hünemeier

**Affiliations:** 1 Departamento de Genética e Biologia Evolutiva, Instituto de Biociências, Universidade de São Paulo, São Paulo, Brazil; 2 Departament de Ciències, Institut de Biologia Evolutiva, Experimentals i de la Salut, Universitat Pompeu Fabra, Barcelona, Spain

**Keywords:** settlement of South America, Andes-Amazonia divide, genetics, native Americans

## Abstract

South America is home to one of the most culturally diverse present-day native populations. However, the dispersion pattern, genetic substructure, and demographic complexity within South America are still poorly understood. Based on genome-wide data of 58 native populations, we provide a comprehensive scenario of South American indigenous groups considering the genomic, environmental, and linguistic data. Clear patterns of genetic structure were inferred among the South American natives, presenting at least four primary genetic clusters in the Amazonian and savanna regions and three clusters in the Andes and Pacific coast. We detected a cline of genetic variation along a west-east axis, contradicting a hard Andes-Amazon divide. This longitudinal genetic variation seemed to have been shaped by both serial population bottlenecks and isolation by distance. Results indicated that present-day South American substructures recapitulate ancient macroregional ancestries and western Amazonia groups show genetic evidence of cultural exchanges that led to language replacement in precontact times. Finally, demographic inferences pointed to a higher resilience of the western South American groups regarding population collapses caused by the European invasion and indicated precontact population reductions and demic expansions in South America.

## Introduction

Human history in South America began as early as 15,000–14,000 years before the present (BP) (Bodner et al. 2012; Rasmussen et al. 2015; Dillehay et al. 2017; Prates et al. 2020). From the initial settlement to the European conquest, different groups of people have dispersed into and within this continent, interacting with the environment and with each other, which has created a complex web of ethnolinguistic and genetic relations that are still poorly understood. Precontact America was extensively populated by numerous and culturally diverse groups (Goldberg et al. 2016) but with contrasting low genetic diversity relative to that of other populations from around the world (Bergström et al. 2020). This ancient cultural diversity is related to geography and several main regions have been historically recognized, such as Mesoamerica, Andes, Pacific coast, western and eastern Amazon, Brazilian savanna and Atlantic coast, Gran Chaco, and Southern Cone.

Present-day Amazonians likely have the highest ethnolinguistic diversity of South America (Hua et al. 2019; Epps 2020), and the Andean cordillera is home to the largest autochthonous population (Davis-Castro 2021). Nonetheless, areas such as eastern Brazil, Gran Chaco, the Pacific coast, and the Southern Cone have suffered extreme reductions in population sizes and cultural diversity, as a consequence of the European invasion and colonization process (Adhikari et al. 2017; Ongaro et al. 2019). Currently, there is a relative overrepresentation of studies on Andean populations when compared with those of Amazonians, the latter being among the least studied populations hitherto. Most of these studies focused on analyzing the genetic diversity of Andean and Pacific Coastal natives, as well as their division with the westernmost parts of the Amazon (Harris et al. 2018; Barbieri et al. 2019; Gnecchi-Ruscone et al. 2019), without properly representing populations from the eastern Amazon, savannas, and southern Brazil. Therefore, there is a substantial lack of genetic information relative to the role of Amazonian and other eastern South American populations in the settlement of the continent and their relationship with other linguistic and geographical groups over time, especially across the Andes-Amazonia divide (Pearce et al. 2020), as well as cultural exchanges within and amongst groups from these diverse regions.

The genetic diversity of South America was most likely shaped by a continuous and dynamic interaction between the autochthonous population and the heterogeneous ecological features of this environment mediated by both cultural and biological evolution. Complex indigenous societies settled in these different South American regions, creating a rich network of exchanges between different cultures and populations, creating one of the most diverse linguistic landscapes in the world. However, the origin of the linguistic and genetic diversity of the people and the roles of the environmental and cultural practices in shaping and maintaining this diversity, in addition to the influence of geography on the distribution of this diversity, are still unknown. Here, we dissected the population and demographic history of the South American natives, illuminating how their genetic diversity is distributed, by analyzing genomic data from populations belonging to 58 different populations from eastern and western Amazon, the Brazilian tropical savanna (i.e., *Cerrado*), the Atlantic coast, the Andes, the Pacific coast, and Mexico (table 1).

**Table 1.** Description of Samples, Groups, and Major Ethnolinguistic/Geographic Groups (also simply referred to as major groups), along with the Labels Used Throughout the Text.

Continental Region	Major Ethnolinguistic Group	*N*	Description
Eastern South America (Brazil)	Jê	45	Jê speakers from northeastern Amazonia and central Brazilian plateau: Xavante (*N* = 27) (Castro e Silva et al. 2021); Xavante (*N =* 11) (Skoglund et al. 2015); Xikrin (*N* = 7) (Castro e Silva et al. 2021)
	Karib	8	Karib speakers from northeastern Amazonia: Apalai (*N* = 4) (Skoglund et al. 2015); Arara (*N* = 4) (Skoglund et al. 2015)
	Tupí	65	Tupí speakers from Amazonia, central-west Brazil and Brazilian Atlantic Coast: Asurini (*N* = 1) (Castro e Silva et al. 2021); Gavião (*N* = 2) (Castro e Silva et al. 2020); Guaraní Kaiowá (*N* = 10) (Skoglund et al. 2015); Guaraní Mbyá (*N* = 4) (Castro e Silva et al. 2020); Guaraní Nãndeva (*N* = 7) (Skoglund et al. 2015); Karitiana (*N* = 13) (HGDP; Patterson et al. 2012); Karitiana (*N* = 4) (Skoglund et al. 2015); Munduruku (*N* = 2) (Castro e Silva et al. 2021); Parakanã (*N* = 3) (Castro e Silva et al. 2020); Surui (*N* = 8) (HGDP; Patterson et al. 2012); Surui (*N* = 4) (Skoglund et al. 2015); Tupiniquim (*N* = 1) (Castro e Silva et al. 2020); Urubu Kaapor (*N* = 3) (Skoglund et al. 2015); Wajãpi (*N* = 2) (Castro e Silva et al. 2020); Zoró (*N* = 1) (Skoglund et al. 2015)
Mesoamerica and northern Mexico (Mexico)	Mayan	21	Mayan speakers from the Mexican Yucatán Peninsula: Maya (*N* = 21) (HGDP; Patterson et al. 2012)
	Mixe-Zoque	12	Mixe from southern Mexico: Mixe (*N* = 10) (Lazaridis et al. 2014); Mixe.DG (*N* = 2) (Skoglund et al. 2015)
	Oto-Manguean	24	Mixtec and Zapotec from southern Mexico: Mixtec (*N* = 10) (Lazaridis et al. 2014); Mixtec.DG (*N* = 2) (SGDP; Mallick et al. 2016); Zapotec (*N* = 10) (Lazaridis et al. 2014); Zapotec.DG (*N* = 2) (SGDP; Mallick et al. 2016)
	Uto-Aztecan	22	Pima and Yaquis from northern Mexico: Pima (*N* = 14) (HGDP; Patterson et al. 2012); Yaquis (*N* = 8) (Barbieri et al. 2019)
Western South America (Bolivia, Colombia, Ecuador, and Peru)	Andes	31	Descendants of indigenous groups from central Peruvian Andes: Huancas (*N* = 5) (Barbieri et al. 2019); LaJalca (*N* = 11) (Barbieri et al. 2019); Luya (*N* = 11) (Barbieri et al. 2019); Utcubamba South (*N* = 4) (Barbieri et al. 2019)
	Arawak	6	Arawak speakers from central Peru (eastern Andean slopes) and southern Colombia: Chamicuro (*N* = 1) (Barbieri et al. 2019); Piapoco (*N* = 5) (HGDP; Patterson et al. 2012)
	Aymara	9	Aymara from southern Peruvian Andes and Aymara descendants from northern Bolivian Andes (Lake Titicaca and adjacent regions): Aymara (*N* = 2) (Barbieri et al. 2019); Bolivian (*N* = 7) (Lazaridis et al. 2014)
	Cahuapanan	1	Shawi from central Peru (eastern Andean slopes): Shawi (*N* = 1) (Barbieri et al. 2019)
	Language Isolate	10	Speakers of language isolates from southwestern Colombia and central Peru (eastern Andean slopes): Cofan (*N* = 4) (Barbieri et al. 2019); Kamentsa (*N* = 4) (Barbieri et al. 2019); Muniche (*N* = 2) (Barbieri et al. 2019)
	Pacific Coast	58	Descendants of indigenous groups from the Pacific Coast of central and northern Peru: Cao (*N* = 10) (Barbieri et al. 2019); Chotuna (*N* = 4) (Barbieri et al. 2019); Chulucanas (*N* = 8) (Barbieri et al. 2019); Eten (*N* = 5) (Barbieri et al. 2019); Narihuala (*N* = 5) (Barbieri et al. 2019); Olmos (*N* = 4) (Barbieri et al. 2019); Paran (*N* = 3) (Barbieri et al. 2019); Sechura (*N* = 3) (Barbieri et al. 2019); Tallan (*N* = 8) (Barbieri et al. 2019); Tumbes (*N* = 8) (Barbieri et al. 2019)
	Quechua	61	Quechua speakers from southern Colombia, northern Ecuador, central Peru (western Amazonia), southern Peruvian Andes and Lake Titicaca region: Inga (*N* = 13) (Barbieri et al. 2019); Kichwa (*N* = 17) (Barbieri et al. 2019); Quechua Cusco (*N* = 3) (Barbieri et al. 2019); Quechua Cusco2 (*N* = 7) (Barbieri et al. 2019); Quechua Cusco3 (*N* = 5) (Lazaridis et al. 2014); Quechua Puno (*N* = 5) (Barbieri et al. 2019); Quechua.DG (*N* = 1) (SGDP (Mallick et al. 2016)); Wayku (*N* = 10) (Barbieri et al. 2019)
	Tupí	10	Tupí speakers from southern Colombia and central Peru (eastern Andean slopes): Kokama Colombia (*N* = 7) (Barbieri et al. 2019); Kokama Peru (*N* = 3) (Barbieri et al. 2019)

Note.—This table includes the continental region of origin, the number of individuals sampled from each major group, and also the label and number of individuals from each indigenous group, along with each respective reference. See supplementary data set S1, Supplementary Material online for a complete description of the samples. Major group labels are based on affiliations to common ethnolinguistic groups and geographic proximity (in cases where no detailed linguistic information was available).

## Results and Discussion

### Patterns of Genetic Ancestry in South America

**Fig. 1.**
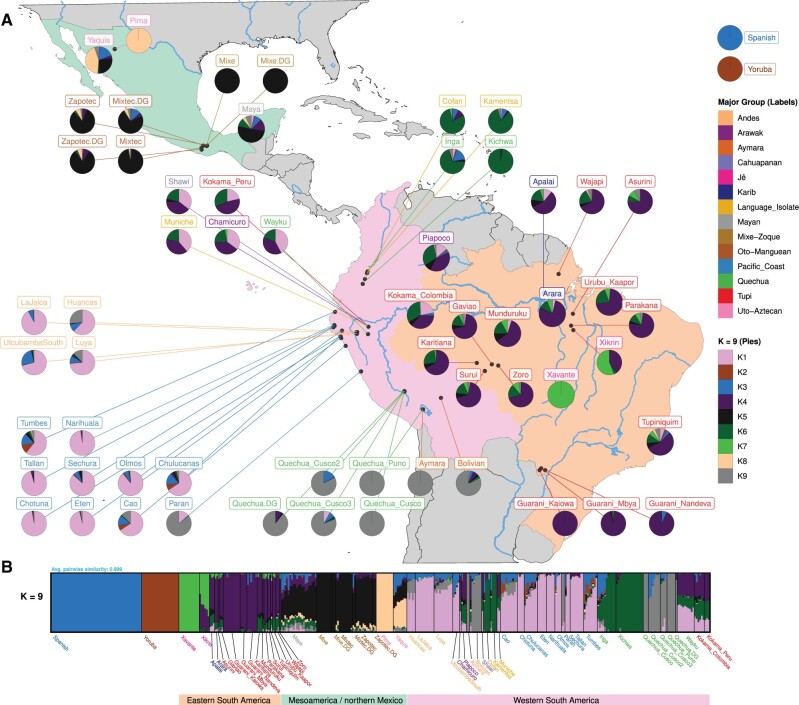
Genetic structure of the Native Americans. An unsupervised admixture analysis with the number of putative ancestry components (*K*) ranging from 2 to 10 was applied to the LD-pruned set of unrelated Native Americans and the results with *K* = 9 are shown here, which is the highest *K* where a consensus was obtained. The complete set of analyses, from *K* = 2 to *K* = 10, is shown in supplementary figures S5 and S6, Supplementary Material online. (*A*) A partial map of the American continent with mean putative ancestry component estimates per group plotted in their approximate sampling locations. (*B*) Bar plot of the individual ancestry component estimates created using PONG (Behr et al. 2016). In (*A*) and (*B*), the name tags and the putative ancestry components are color coded as indicated by the legends on the right (major group affiliations as in table 1). Finally, the three main continental regions are indicated by color shade in (*A*) and colored bar at the bottom in (*B*): Mesoamerica and northern Mexico in light green, western South America in pink, and eastern South American in beige.

First, using ADMIXTURE we estimated the proportion of African and European components present in the admixed individuals (we use a threshold of more than 1% inferred non-Native American ancestry to identify those with postcontact admixture), and the proportions per individual varied from 0% to 22% (mean 1%) and 0% to 44% (mean 6%) for the African and European ancestry components (supplementary fig. S1, Supplementary Material online), respectively. Additionally, we demonstrated that these component proportions are not significantly correlated with the geographic locations of these indigenous groups, represented by latitude and longitude, as inferred with a linear regression model (supplementary fig. S2, Supplementary Material online). Then, we began to unravel the relationship among the contemporary indigenous groups (supplementary figs. S3 and S4, Supplementary Material online) and to examine the influence of geographic and cultural factors (here we use the term culture to refer to language and ethnicity, which are proxies for other cultural characteristics) on the distribution and structure of genetic diversity. By analyzing the complete set of unrelated Native Americans, we identified nine clusters with moderate to high similarity based on genetic profiles, as follows: 1) northern Mexico, 2) southern Mexico, 3) northern Andes, 4) southern Andes, 5) Pacific Coast and central Andes, 6) western Amazonia, including the eastern Andean slopes, 7) Karib and Tupí speakers from central and eastern Amazonia and the Atlantic Coast, 8) Jê speakers from eastern Amazonia and the central Brazilian plateau, and 9) Guaraní from Central-West Brazil (also Tupí speakers) (fig. 1 and supplementary figs. S5 and S6, Supplementary Material online). Notably, when only the unadmixed (i.e., nonadmixed) and unrelated individuals were analyzed, we also identified four primary genetic similarity clusters in the Amazonian and savanna regions (matching the previous clusters 6–9). The most prominent genetic structure separated Jê speakers from the other Native Americans (supplementary figs. S7 and S8, Supplementary Material online). The second group to be differentiated was the southern branch of Tupí speakers, Guaraní. The remaining non-Jê/non-Guaraní component was then subdivided into a predominant component in western South America and Mesoamerica and another predominant component in eastern South America, basically dividing central/eastern and western Amazonia (fig. 1).

These results suggest no hard genetic divide between the Andes and Amazonia or between their putative areas of influence, classified here in western and eastern South America, respectively. Our data set encompassed groups from Bolivia, Colombia, Ecuador, and Peru, representing western South America, whereas eastern South America was represented by groups from Brazil (Amazon, Savanna, Atlantic coast; table 1). This permeability between the two regions was shown by the shared ancestry components between the Andean and Pacific coastal populations and the western Amazonian populations, especially those located in the eastern Andean slopes (fig. 1 and supplementary figs. S5 and S6, Supplementary Material online), corroborating previous genetic studies (Harris et al. 2018; Barbieri et al. 2019). The western Amazonian groups situated more closely to the Andes exhibited a mixed profile, which suggests that the Andean lineages could have been introduced in the western Amazonia via gene flow, as already observed in the past (Gnecchi-Ruscone et al. 2019). This scenario of an asymmetrical contribution of ancestry from Andeans to western Amazonians is also consistent with the expectations drawn from our demographic inferences, which showed that Andeans had much greater population sizes than Amazonians (see “Population Dynamics and Demography” section) because gene flow is more likely to originate from and less likely to have significant contributions in larger populations, simply by chance.

**Fig. 2.**
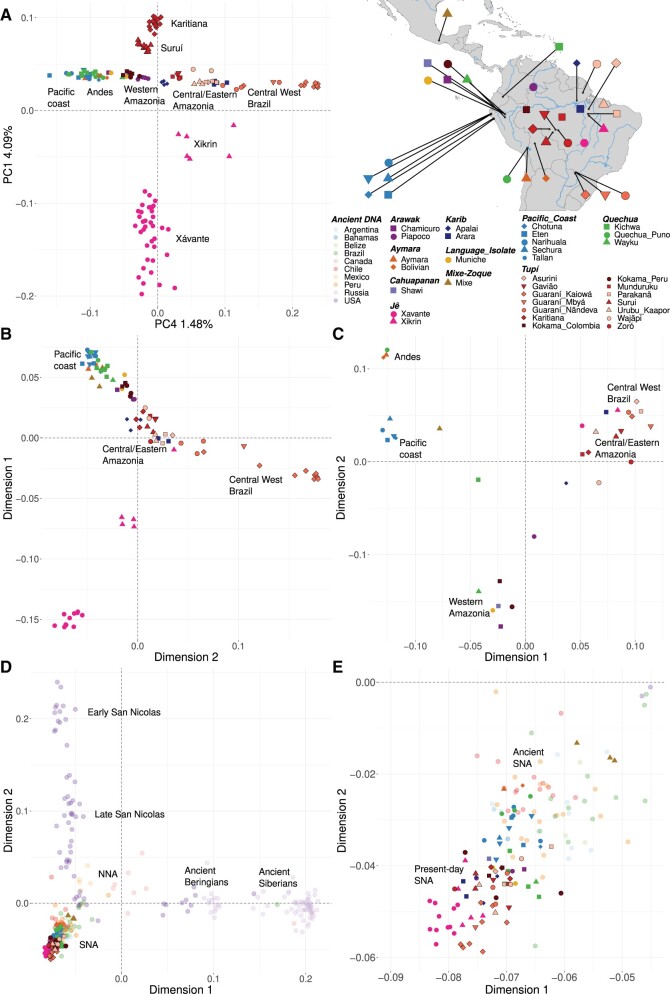
Global patterns of ancestry and genetic affinity among present-day and ancient Native Americans. (*A*) A PCA was applied to the LD-pruned subset of unadmixed and unrelated Native Americans and here we show PC1 and PC4 because it captures the longitudinal cline. The complete set of analyses is shown in supplementary figure S9, Supplementary Material online. Using the same data set, we estimated genetic distances as 1 − outgroup *F*_3_ (Mbuti; *Y*, *Z*), where *Y* and *Z* are any indigenous group or individual. MDS was then applied to the matrices of pairwise genetic distances. (*B*) MDS of the population-wise genetic distances matrix. (*C*) MDS of the individual-based genetic distances matrix. The complete set of *F*_3_ statistics is presented in supplementary data set S5A and B, Supplementary Material online. Finally, ancient DNA samples from across the whole American continent and Siberia were included in the analysis, and the pairwise genetic distances were calculated as 1 − outgroup *F*_3_ (Mbuti; *Y*, *Z*), where *Y* and *Z* are any present-day and ancient individuals. (*D*) MDS of the complete data set. (*E*) Southern Native Americans (SNA) in more detail. The complete set of statistics is presented in supplementary data set S5C, Supplementary Material online. The legend at the top right shows the symbol and color used for each present-day group (major group affiliations as in table 1) or the country of origin of each aDNA sample, and the map indicates the approximate location of each group.

The inferred genetic structure pattern, along with the principal component analysis (PCA) (fig. 2*A* and supplementary fig. S9, Supplementary Material online), also indicated that the geographic distribution plays an important role in shaping the genetic similarity of South American groups. Hence, this relationship between genetic variation and geography is also evidenced by the PCA, where at least one of the principal components was correlated at some degree with longitude (fig. 2*A* or supplementary fig. S9*A*, Supplementary Material online: PC4 ∼ longitude: *R*^2^ = 0.3962 and *P* value = 2.2e–16; supplementary fig. S9*B*, Supplementary Material online: PC1 ∼ longitude: *R*^2^ = 0.5905 and *P* value = 2.2e–16), although some groups are outliers (Xavante, Xikrin, Karitiana, and Suruí). This result suggests that the genetic variation is more structured in the longitudinal direction, which is a rather unexpected result, as environmental and climatic conditions tend to vary less in this axis, thus facilitating population movement. However, the South American continent might be an exception, considering the amount of environmental and climatic variation in a longitudinal axis from the Pacific to the Atlantic coast, mainly owing to the influence of the Andes on the distribution of climatic conditions. Our results on computed matrices of genetic (estimated as 1 − outgroup *F*_3_) and geographic distances (great circle distances) between all pairs of individuals indicated (*P* ≤ 2.2e–16) that populations more closely located tend to be more genetically similar and are therefore compatible with an isolation-by-distance model. Additional evidence came from the multidimensional scaling (MDS) of individual-based (fig. 2*B*) and population-wise (fig. 2*C*) genetic distances (1 − outgroup), which also revealed the existence of a genetic gradient aligned with the west-east axis, though the population-wise analysis indicated additional substructure (clusters of groups from the Andes, the Pacific coast, Western Amazonia, Central/Eastern Amazonia, and Central-West Brazil; fig. 2*C*). However, the influence of geographic distribution and environmental and cultural diversity is difficult to disentangle, as groups speaking the same language and sharing a common culture tend to live in proximity and settle in the same kinds of environments. To evaluate the influence of ethnolinguistic diversity on the genetic variation, we used AMOVA (Excoffier et al. 1992) to examine the existence of genetic structure among individuals, ethnic groups, and linguistic groups (i.e., major groups; table 1). We detected a significant genetic structure in all hierarchical levels, with the exception of the variation among samples within a population (*P* = 0.148; supplementary table S1, Supplementary Material online); therefore, the existence of random mating between individuals from the same populations cannot be rejected. At the same time, genetic differentiation between populations, from distinct ethnic groups (*P* = 0.001; supplementary table S1, Supplementary Material online), and between linguistic groups is inferred (*P* = 0.004; supplementary table S1, Supplementary Material online), represented 36.51% and 16.22% of the variation (discounting within-individual variation), respectively. These results indicate that ethnolinguistic diversity plays a significant role in shaping the genetic variation and also that individuals are significantly differentiated between populations but not within them, a pattern compatible with a history in which most populations would be relatively small and isolated.

**Fig. 3.**
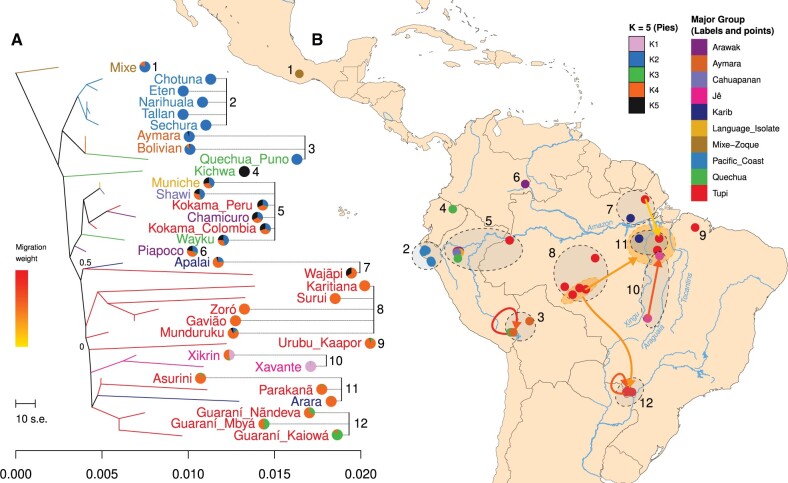
Population diversification patterns reflect the geographic distribution. Using the LD-pruned subset of unadmixed and unrelated Native Americans, (*A*) a maximum likelihood (ML) tree was estimated based on pairwise population covariance using Treemix (Pickrell and Pritchard 2012), and gene flow events were progressively modeled between the branches of the ML tree with the poorest fit. The model likelihood reaches a plateau at six gene flow events; therefore, we additionally present these gene flow events (exclusively in *B*). Using the same data set, we also performed an unsupervised admixture analysis and we present the results with *K* = 5 as pie charts at the right side of each group in the ML tree. The complete set of analyses is shown in supplementary figures S7 and S8, Supplementary Material online. (*B*) Group geographic locations are indicated as points on a map, which along with group labels on the ML tree (*A*) are color coded to indicate affiliation to the major groups (table 1). Finally, we also cross-reference the groups on the ML tree (*A*) to their geographic locations on the map (*B*), as well as gene flow events (color-coded arrows), inferred in the model with six gene flows (likelihood plateau; supplementary fig. S10, Supplementary Material online), indicating their direction (arrowheads) and intensity (color coded).

Furthermore, the historical relationships among populations inferred with Treemix (Pickrell and Pritchard 2012) broadly recovered the same clusters observed in the genetic structure analyses (fig. 1), namely the Pacific Coast, the southern Andes, and the Kichwa, which remains isolated as the sole representative of a northern Andean cluster in this analysis, another division between western and central/eastern Amazonia, and the remaining Brazilian samples clustered with the latter (fig. 3). The eastern South American/Brazilian branch was subdivided into two main regions, north (with Apalai and Wajãpi) and south of the Amazon River, the latter being further subdivided into central Amazonia (i.e., Munduruku) and the Madeira-Guaporé region (Gavião, Karitiana, Suruí, and Zoró); finally, Xavante and Urubu-Kaapor presented with no further clustering. We also found a cluster delimited by the Xingu River in the west and by the Araguaia and Tocantins rivers in the east (Asurini, Parakanã, and Xikrin), including Arara, located in the Xingu River near the west margin (fig. 3). The genetic structure patterns suggested that rivers might have acted as deterrents to gene flow, as some divisions seemed to coincide with their courses. Additionally, the cluster of Guaraní groups was differentiated from the others (fig. 3) located in Central-West Brazil. Most importantly, this result also indicates that the pattern of population diversification aligns with a west-east axis, possibly tracing back to the initial settlement events and that geographic distance and environmental diversity play important roles in shaping genetic similarities among groups.

We also inferred putative gene flow events using Treemix by fitting an increasing number of gene flow events until a model likelihood plateau was reached with six events (color-coded arrows in fig. 3 and supplementary fig. S10, Supplementary Material online). Of note, most inferred gene flow events occurred between eastern South Americans, especially among Tupí-speakers as follows: 1) Wajãpi to Asurini; 2) Guaraní Kaiowá to Guaraní Mbyá; 3) the inferred most recent common ancestor (MRCA) of Tupí-Mondé (Surui, Gavião, and Zoró) to Guaraní Mbyá; 4) Zoró to the inferred MRCA of northeastern Amazonians located in the southern margin of the Amazon River (Arara, Parakanã, Xikrin, and Asurini); and 5) Xavante to Xikrin (both Jê speakers). We also detected one gene flow event from Quechua of the southern Peruvian Andes to a group of Bolivians (with Aymara ancestry).

### Pre-Columbian Interactions: Cultural Exchange and Admixture

The Tupí linguistic group currently has the largest number of speakers among South American lowlanders. In addition to this large proportion of speakers, the group’s wide geographical distribution draws attention, with populations ranging from the Atlantic coast (Tupiniquim) to the northwest boundary between the Amazon forest and the Andes (Kokama) and to the region close to the borders between Brazil, Argentina, and Paraguay (Guaraní), with more than 5,000 km in the maximum expansion range. During the Tupí expansion (Noelli 2008), the Tupí came into contact with different people from other ethnolinguistic origins, and these interactions were certainly very diverse in the way that they developed. Depending on both the cultural characteristics of the Tupí and of those with whom they interacted, different degrees of cultural exchange and admixture took place. Thus, here, we studied the Kokama and the Guarani, two extremes of the Tupí Expansion, as examples of how Pre-Columbian interactions produced quite different outcomes and shaped the landscape of genetic and cultural diversity.

**Fig. 4.**
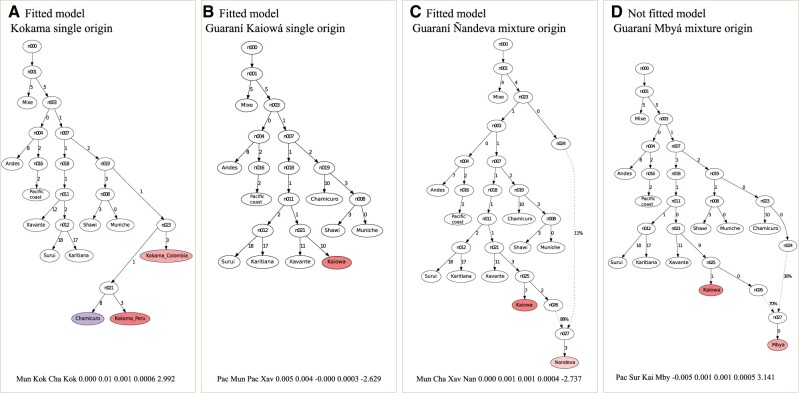
Evidence of Pre-Columbian cultural exchange and admixture between South American natives. We leveraged the unadmixed and unrelated subset of Native American groups to model the possible ancestral contributions to the Kokama and Guaraní ethnolinguistic groups. (*A*) Best fitted model for the Kokama group (from Peru and Colombia)—single origin. (*B*) Best fitted model for the Guaraní Kaiowá group—single origin*.* (*C*) Best fitted model for Guaraní Nãndeva group—mixed origin. (*D*) Best fitted model for Guaraní Mbyá group—mixed origin.

Interestingly, the Kokama groups (from Colombia and Peru) presented genetic ancestry profiles much more similar to populations from western Amazonia, especially those from the Loreto region located close to the eastern Andean slopes (i.e., Chamicuro, Muniche, Shawi, and Wayku), rather than to other Tupí-speaking groups, as shown by ADMIXTURE, PCA, and Treemix (figs. 1, 2A, and 3). Additionally, admixture graph models showed a good fit for the one-way model or single-origin model for the Peruvian Kokama, placing them as a sister branch of the Chamicuro, an Arawak-speaking group (fig. 4). Furthermore, the Colombian Kokama can be modeled as a basal group to the Peruvian Kokama and Chamicuro (fig. 4*A* and supplementary fig. S11*A*, Supplementary Material online). Other evidence came from the MDS of pairwise genetic distances (1 − outgroup *F*_3_), in which both Kokama groups were clustered with western Amazonians (fig. 2*B* and *C*), rather than with the other Tupí, and also from *F*_4_ statistics, where we detected an excess affinity among both Kokama groups (supplementary fig. S12, Supplementary Material online), as well as between them and the Arawak speakers, which was especially strong when comparing Peruvian Kokama and Chamicuro (median Z value < −3 in supplementary fig. S12, Supplementary Material online). This genetic similarity could theoretically be the consequence of the recent population influx from rural areas into large urban centers in the Andean region, which resulted in homogenization of the local genetic diversity (Tarazona-Santos et al. 2001). Meanwhile, this could partially explain this pattern in the Peruvian Kokama, but it cannot explain why the Colombian Kokama also presented genetic profiles very similar to those of the Arawak-speaking groups and the other western South American populations, even though they are located hundreds of kilometers away.

This striking result aligns with a previous hypothesis about the origins of present-day Kokama. According to this interpretation, the Kokama were a non-Tupí-speaking people who went through a process of language replacement due to contact with speakers of Tupí-Guaraní (Noelli 2008; Michael 2014), likely during the period of the Tupí Expansion event around 3,000–2,000 BP. In that regard, a combination of linguistic, historical, and ethnographic analysis (Michael 2014) favored a Pre-Columbian origin for the Kokama group. According to this time frame, this language replacement would not be an outcome of the contact with Europeans and the consequent disruption of indigenous societies. Therefore, our results on the marked genetic similarity between Kokama and the Arawak speakers added to the latter’s unique cultural characteristics (e.g., exogamy, high mobility, and the territorially expansive nature of the Arawak; Hornborg 2005) and supports the Kokama as former Arawak-speakers (or closely related groups) that adopted a Tupí-Guaraní language, likely due to the Tupí expansion.

At the other end of the Tupí expansion, on the border among Argentina, Brazil, and Paraguay, are the Guaraní people, currently representing most Tupí speakers, who were divided into three ethnic groups, Kaiowá, Mbyá, and Ñandeva. Previous studies have shown that the Pre-Columbian admixture with Gran Chaco, Mesoamerican, or other related sources probably contributed to the formation of this southern Tupí branch (Reich et al. 2012; Gnecchi-Ruscone et al. 2019; Castro e Silva et al. 2020). Accordingly, we used Admixture Graph modeling to infer the population history of the Guaraní group, and several models presented a good fit to the genetic data, considering all possible combinations of single or mixture models. On one hand, the single origin of Guaraní Kaiowá (fig. 4*B*) as a sister group of Xavante (Jê speakers) indicated a closer relationship between the ancestors of the two groups in comparison to that with the Amazonians, here represented by Suruí and Karitiana. On the other hand, the Guaraní Ñandeva presented several models with good fit (fig. 4*C* and supplementary fig. S11*B*–*I*, Supplementary Material online). Among these possibilities, Ñandeva could be fitted as an admixed population resulting from a major Tupí-Guaraní ancestral component and a minor component related to early branches of South American groups (e.g., Mixe, Andes, Pacific Coast, eastern Andean slopes groups, or basal South Americans; fig. 4*C* and supplementary fig. S11*B*–*I*, Supplementary Material online). We were not able to model Guaraní Mbyá into a well-fitted model. The lowest maximum Z of the mixture model was 3.141 (fig. 4*D*) in a pattern very similar to the population history models inferred for Guaraní Ñandeva.

We were likely unable to fit the Guaraní Mbyá into a model, as well as to distinguish a clear best-fitted model for Guaraní Ñandeva, owing to significant contributions from Gran Chaco lineages or other closely related groups not represented in our data set. This admixed origin of Guaraní was first inferred by Reich et al. (2012) as a mixture of ancestral lineages of sister branches of the present-day Wichi (Gran Chaco) and Suruí (Amazonia); later, this inference of an admixture event was also reproduced by others (Gnecchi-Ruscone et al. 2019). Additionally, we previously detected an ancestry contribution from a Mesoamerican source into the Guaraní branch (Castro e Silva et al. 2020), which likely represents the same event, as we also lacked samples from the Gran Chaco and other adjacent regions for that specific analysis. These results show how the interaction between different groups, as well as the resulting cultural exchanges and admixture, have significantly influenced the current pattern of genetic and cultural diversity among Native American groups.

### Genetic Affinities amongst Present-Day South American Natives

We then tested the existence of excess allele sharing among particular Native American groups by calculating the *F*_4_ (Mbuti, *X*, *Y*, *Z*) for every combination of *X*, *Y*, and *Z* Native American test groups (supplementary fig. S12, Supplementary Material online). Using this approach, we detected continental-wide excess affinity patterns of interest, which largely coincided with the results from the genetic structure analyses, as follows: 1) an excess affinity between Kokama and Arawak speakers; 2) a ubiquitous high affinity between the groups from the Madeira-Guaporé region (Amazonia—Rondonia state); 3) a pattern of high affinity amongst Guaraní groups (Kaiowá, Mbyá, and Ñandeva); 4) Jê speakers presented high affinities with one another; 5) finally, groups from the Pacific coast and the Andes regions presented high and almost exclusive intra-regional affinity.

**Fig. 5.**
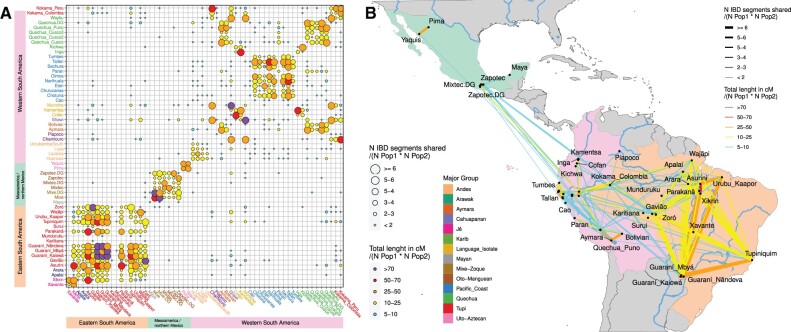
Network of Pre-Columbian IBD sharing among present-day Native American groups. The IBD genomic segments were identified based on the phased data subset of unrelated Native Americans, then these segments were filtered to select only those inferred to be in genomic regions of Native American local ancestry. Segments shorter than 2 cM were removed and pairwise connections with less than 5 cM shared on average were also not considered. Here, we present the results obtained using IBD segments with at most 8.4 cM of length, which approximately correspond to those that originated in the Pre-Columbian period (before 1500 CE). The average number of IBD segments (color) and the average length of IBD in cM (size), are shown as a matrix (*A*) and as a network on a map (*B*). The classification of populations into major groups (table 1) is also color coded, as indicated in the legend at the center (axes labels in *A*). The three main continental regions are indicated by a set of colored bars at the left and the bottom of *A*, matching the same colors used in the map regions in (*B*). The intrapopulation IBD is shown in the diagonal in (*A*). Some group labels are shown in (*B*) for reference. For the patterns of IBD sharing in the colonial and recent periods, see supplementary figure S10, Supplementary Material online. The complete set of IBD segments inferred are presented in supplementary data set S4, Supplementary Material online.

Additionally, the genetic affinities among the groups were also examined through the identification of identical by descent (IBD) segments present in genomic regions of Native American local ancestry. We adapted the approach used by Barbieri et al. (2019), restricting this analysis to connections between groups that share a total average of at least 5 cM of IBD, to remove ubiquitously shared short fragments and spurious connections. We also removed related individuals to avoid biased estimates of IBD, and the selected segments were grouped into three length categories to focus on different approximate periods (Baharian et al. 2016; Harris et al. 2018) as follows: 1) Pre-Columbian period ∼< 1500 CE (segments ≤ 8.4 cM) (fig. 5); 2) colonial period ∼1500–1850 CE (8.4 cM < segments ≤ 28 cM) (supplementary fig. S13*A*, Supplementary Material online); 3) the recent period ∼1850–present CE (28 cM > segments) (supplementary fig. S13*B*, Supplementary Material online).

The results showed a widespread network of IBD sharing formed in the Pre-Columbian period, with a much higher number and intensity of connections among groups within a few main areas (which largely corresponded to the clusters of genetic similarity identified previously; e.g., figs. 1, 2C, and 3, and supplementary fig. S12, Supplementary Material online) as follows: the Pacific coast; the southern, central, and northern Andes; northern and southern Mexico; and especially central and eastern Amazonia, along with other Brazilian regions with less intense connections among these main areas (fig. 5). This observation also contrasts a hard Andes-Amazonia divide model (Pearce et al. 2020), as western Amazonians showed similar connectivity with both the Andeans and the central and eastern Amazonians, suggesting them to be intermediaries or transitional groups, corroborating the results from the genetic structure analyses (fig. 1). In addition, for IBD originating in the colonial period (supplementary fig. S13*A*, Supplementary Material online), as well as in the more recent period (supplementary fig. S13*B*, Supplementary Material online), IBD sharing was even more restricted to these main areas with very few inferred long-distance connections. These observations also indicated that the genetic diversity of groups from different geographical areas (e.g., Pacific coast, central Andes, and eastern Amazon) evolved more independently and possibly in distinct directions, both biologically and culturally. This is because they presented fewer genetic connections, indicative of fewer interactions and more distant common ancestors in time, along with the fact that they have withstood different geographic, climatic, ecological, and cultural forces in their recent and ancient histories.

### Present-Day South American Substructure Recapitulates Deep Macroregional Ancestries

Furthermore, to assess the influence of genetic continuity on the distribution of present-day genetic diversity, our approach aimed to detect the existence of excess allele sharing between present-day Native American groups and ancient individuals, concerning other present-day indigenous groups in the Americas. To accomplish this, we computed *F*_4_ (Mbuti, *X*; *Y*, *Z*), where *X* is any ancient sample and *Y* and *Z* are any present-day Native American group. The complete set of statistics was then summarized as the number of highly significant tests (*Z* > 4) grouped by pairs of *X* ancient individuals and *Z* present-day groups summed over all *Y* present-day groups (supplementary fig. S14, Supplementary Material online). Whereas the specific pattern of affinity changed between different sample ages and locations, our findings demonstrated a general excess affinity between ancient and present-day groups from the same or adjacent regions or the most mutually accessible regions (e.g., Amazonia and Caribbean Islands; Pacific coast and the Andes). This finding suggests at least some level of genetic continuity within large continental areas, which is arguably expected due to 1) the fact that even the most extensive population movements (after the initial settlement of the continent) were mostly restricted to specific continental areas (e.g., central and northern Andes in the case of the Inca expansions; present-day Brazilian territory in the case of the Tupí Expansion) and 2) these movements often lead to partial admixture between the newcomers and the local populations, therefore leading to some level of genetic continuity. Although these differences in genetic affinity with ancient samples could be small, they would be significant enough to be detected in comparisons between present-day groups located in distant continental regions. Precisely, the broad patterns indicated an excess affinity between ancient samples from Bolivia, Chile, and Peru with present-day southern Andean populations (Aymara and Quechua) and in the case of some Argentinian and Peruvian ancient samples, to present-day Pacific coastal groups (supplementary fig. S14, Supplementary Material online). However, ancient samples from eastern South America (Brazil), Central America (Belize), and the Caribbean islands (Bahamas) tended to exhibit higher affinity with present-day groups from Brazil, more specifically with speakers of Tupí, Jê, and Karib languages (supplementary fig. S14, Supplementary Material online).

We also analyzed the global pattern of genetic affinities among present-day Native Americans and ancient individuals from across the whole American continent and Siberia. For this, we estimated the genetic distances (1 − outgroup *F*_3_) between all pairs of individuals and applied an MDS to the matrix of pairwise genetic distances. The analysis identified the most distinctive groups (fig. 2*D*, Dimension 1), with ancient Siberians and Ancient Beringians (AB) separated from the Northern Native Americans (NNA) and Southern Native Americans (SNA) (Reich et al. 2012; Rasmussen et al. 2014; Raghavan et al. 2015; Moreno-Mayar et al. 2018; Posth et al. 2018) and with all present-day Native Americans clustered with the latter. Ancient individuals from the San Nicolas Island were outliers in the MDS (fig. 2*D*, Dimension 2), likely a consequence of extreme genetic drift effects, as expected for small and isolated populations living on islands. With a closer look at the SNA, a cline of genetic differentiation was detected, as in previous analyses (fig. 2*A*–*C*). However, this time, the gradient broadly progressed from ancient samples from the North to Central and South America (fig. 2*E*, top right corner), followed by present-day groups from Mesoamerica (Mixe), the Pacific Coast, Andes, and Amazonia, and ending with Jê speakers from the central Brazilian plateau and Guaraní groups from Central-West Brazil (fig. 2*E*, bottom left corner). Accordingly, if only the values of present-day individuals obtained in this MDS were considered and linear regression models were applied between the first and second dimensions of the MDS with the longitude or latitude of each individual, the models “lm(Dimension 1 ∼ Longitude)” and “lm(Dimension 2 ∼ Longitude)” presented estimated *R*^2^ values of 0.4882 (*P* = 3.195e^−14^) and 0.5274 (*P* = 1.041e^−15^), whereas the models “lm(Dimension 1 ∼ Latitude)” and “lm(Dimension 2 ∼ Latitude)” exhibited estimated *R*^2^ values of 0.2830 (*P* = 6.855e^−08^) and 0.2904 (*P* = 4.348e^−08^), respectively. Once again suggesting the relationship between genetic variation and geography, especially with the longitudinal distribution.

### Population Dynamics and Demography

**Fig. 6.**
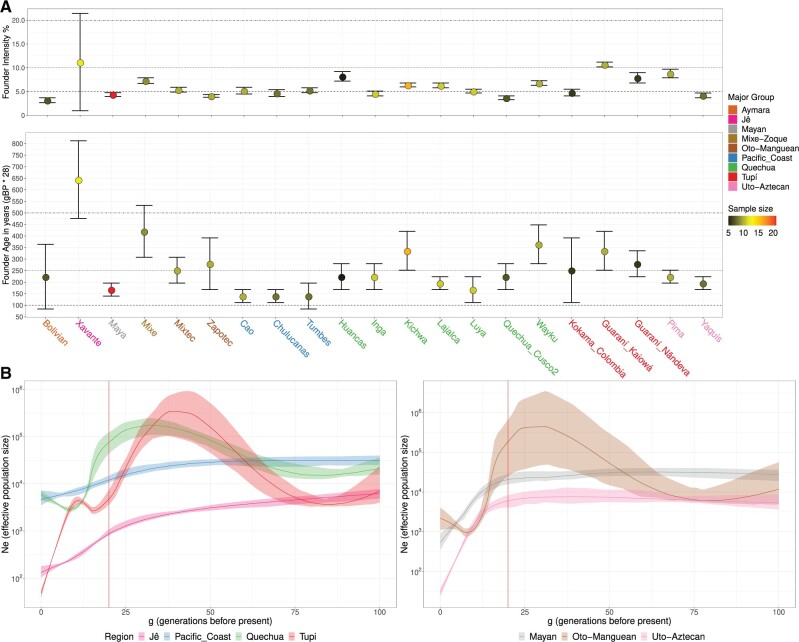
The postcontact population collapse and Native American effective population size (N_e_) histories. (*A*) We applied the ASCEND (Tournebize et al. 2020) method to every Native American group with more than 5 unrelated samples (and also to some clusters of groups to reach the minimum sample size of 5, see supplementary fig. S15, Supplementary Material online), and we also selected the groups with an estimated FA lower than 1000 BP. In *A*, the top panels depict the FI, and the bottom panels show the mean estimate of the FA for each indigenous group. For each group, the estimated FI and FA are shown along with their associated 95% confidence interval. The sample size is color coded on the points and the affiliations with major groups are indicated in the group label IDs at the *x*-axis, both indicated in the legend. In the top panels, the *y*-axis indicates the FI percentage and in the bottom panel, the *y*-axis shows the estimated FA calculated as: “x” generation before present (gBP) * 28 years per generation = “y” years before present (BP). (*B*) The IBD genomic segments were identified with the phased data set of Native American groups, followed by a selection of the segments inferred to be in genomic regions of Native American ancestry. The complete set of IBD segments was separated into subsets of major groups (table 1) from South America (*B* left) and Mesoamerica/Northern Mexico (*B* right), and then each set was used to infer the *N*_e_ history of each specific major group. The ancestry-specific *N*_e_ values are coded in the *y*-axis (log scale) and indicated by a line for each generation before the present (gBP) depicted in the *x*-axis. The shaded areas show a 95% bootstrap confidence interval for each major group. The vertical red line indicates 20 gBP (approximately 1500 CE) and therefore the time of the first contact with Europeans. Here, we show the results of IBDNe using the parameter filtersamples = “false,” alternatively the results produced with the parameter filtersamples = “true” are shown in supplementary figure S16, Supplementary Material online.

Lastly, contact with the Europeans and the colonization of the American continent led to massive depopulation of the indigenous people caused by the introduction of new diseases (e.g., smallpox), enslavement, warfare, disruption of subsistence strategies, and forced displacement from territories, among other processes (Montenegro and Stephens 2006; Adhikari et al. 2017). To evaluate the effect of these population bottlenecks on the genetic diversity of indigenous populations of the Americas, inferring when they occurred and how strong they were, we applied ASCEND (Tournebize et al. 2020) to all populations with more than five unrelated individuals (fig. 6*A*). Some populations were also analyzed in clusters of speakers of languages from the same linguistic families (to reach the minimum sample size required for the analysis). For each population and cluster, the Founder Intensity (FI; an estimate of the genetic drift strength caused by a bottleneck) and Founder Age (FA; inferred date of the bottleneck) were estimated, along with the associated 95% confidence intervals (Tournebize et al. 2020).

Most groups produced FI estimates concentrated at approximately 5–10% and FA estimates less than 500 BP or approximately 18 generations before present (gBP; considering 28 years per generation; fig. 6*A* and supplementary fig. S15, Supplementary Material online). The median FA and median FI for the groups were 8 gBP (or ∼224 BP) and 5.3%, respectively. The highest inferred FI estimates for groups were obtained for a few Pacific coastal groups (Narihuala: 15.4%; Tallan: 12.5%) and a few groups speaking Jê (Xikrin: 15.3%; Xavante: 11.2%), Tupí-Guaraní (Guaraní_Kaiowá: 10.7%), Uto-Aztecan (Pima: 8.8%), Quechua (Huancas: 8.2%), and Mixe-Zoque (Mixe: 7.3%) languages. The lowest FI estimates were obtained for some Mexican (Zapotec, 4.1%; Yaquis, 4.2%; Maya, 4.4%) and Southern Andean (Bolivians: 3.2% and Quechua_Cusco2: 3.7%) groups. Most of the inferred population bottleneck dates (i.e., FA) occurred in postcontact times, which is in line with the historical records of mass extermination of indigenous people initiated with the arrival of Europeans.

In addition, to estimate the population sizes and evaluate how they have changed over time for the ancestors of the present-day groups from these diverse regions, we also leveraged the IBD segments by applying IBDNe (Browning et al. 2018b) to infer the effective population size histories. This analysis was conducted by sub-setting the IBD segments into the major ethnolinguistic/geographic regions (table 1) and selecting only those with at least 20 samples (to minimize the effects of low sample sizes in the analysis), thereby maintaining the data set with the following clusters: Pacific Coast, Quechua, Tupí, Jê, Maya, Oto-Manguean, and Uto-Aztecan. These subsets were then used to infer the Native American ancestry-specific *N*_e_ history of these groups by including only the IBD segments identified in the genomic regions of Native American local ancestry, as inferred by RFMix (Maples et al. 2013).

As expected, the historical *N*_e_ of eastern South Americans was smaller (median, minimum, and maximum of 9,280, 867, and 90,200, respectively) than that of western South Americans, which showed the highest *N*_e_ (median, minimum, and maximum of 28,000, 7,380, and 143,000, respectively) in the last 100 gBP, whereas Mesoamerica and northern Mexico had values similar to those of western South America (median, minimum, and maximum of 26,500, 805, and 32,700, respectively; fig. 6*B* and supplementary fig. S16, Supplementary Material online). The effective population size estimates also provided evidence of the effect of the European invasion and colonization on the Native American people, with an average reduction of 90.38% in the last 20 gBP. The greatest impact was estimated for Mexican groups, with a 99.49% effective population size decline among Uto-Aztecans, 99.34% among Oto-Mangueans, and 97.31% among Maya, only being equated to an estimated reduction of 98.93% for Tupi, followed by Quechua with a 95.60% decrease, and finally, a significantly smaller reduction of 83.01% for the Jê was inferred, which was much lower on the Pacific coast at 59.03%. These results were very similar to those obtained in the ASCEND analysis when the average FI for each linguistic group was considered (supplementary fig. S17, Supplementary Material online). Among Mexican natives, the highest values were estimated for the Mixe-Zoque (7.3%; not included in the IBDNe analysis) and Uto-Aztecan (6.5%), followed by those for the Oto-Mangueans (4.75%) and Mayans (4.4%), whereas in South America, the highest estimates were observed for the Jê (11.2%), followed by the Tupí (7.8%) and then the Quechua (5.87%) and the Pacific coast (5.07%), and finally the Aymara (3.2%; also not represented in the IBDNe analysis). Taken together, these results show that the impact was differently distributed across the continent, pointing to the conclusion that on average, eastern South America and Mesoamerica were moderately more impacted by the European invasion and colonization process.

Looking at the inferred effective population size histories of the major ethnolinguistic/geographic groups (table 1) represented here (those with more than 20 individuals), it is noticeable that most groups still have a *N*_e_ of at least ∼10^3^ (Mayan, Oto-Manguean, Andes, Pacific Coast, Quechua); however, some of them showed a steep decline in *N*_e_, even going below ∼10^2^ (Uto-Aztecan, Jê, and Tupí) (fig. 6*B* and supplementary fig. S16, Supplementary Material online). Notably, an additional bottleneck was inferred for the Tupí, starting at ∼37 gBP (∼1036 BP) before contacting Europeans and happening up to ∼15 gBP (∼420 BP). This population decline agrees with a previous report based on Amazonian fossil pollen analysis, which pointed to an expansion of the Amazon forest, a proxy for human population decline, at approximately 300–600 years before the European arrival (Bush et al. 2021). However, the inferred time for the population collapse might be influenced by differences in the generational interval; a new study (Coll Macià et al. 2021) inferred that South American lowlanders have the shortest generation intervals in the Americas, which could ultimately translate into older dates for inferences based on the number of generations, including the inference of older population collapse dates from the size distribution of IBD segments.

Our results also showed substantial population expansions of some of these groups in the precontact period, especially between ∼75 and ∼20 gBP. In Mesoamerica, the speakers of Oto-Manguean languages expanded from a *N*_e_ of 10^4^ to almost 10^6^. In South America, especially Tupí speakers and the Andean populations presented with an increase of at least two orders of magnitude (fig. 6*B* and supplementary fig. S16, Supplementary Material online). The Tupí speakers, specifically, exhibited a *N*_e_ increase of 98.92%, which went from ∼75 to ∼35 gBP (approximately between 2100 and 1000 BP), thereby partially overlapping with the hypothesized period of the Tupí Expansion, which would have started between 3000 and 2000 BP (Noelli 2008). This result suggests that Tupí-speaking groups would have gone through population growth during this time, which is one of the proposed drivers of their territorial expansion.

**Fig. 7.**
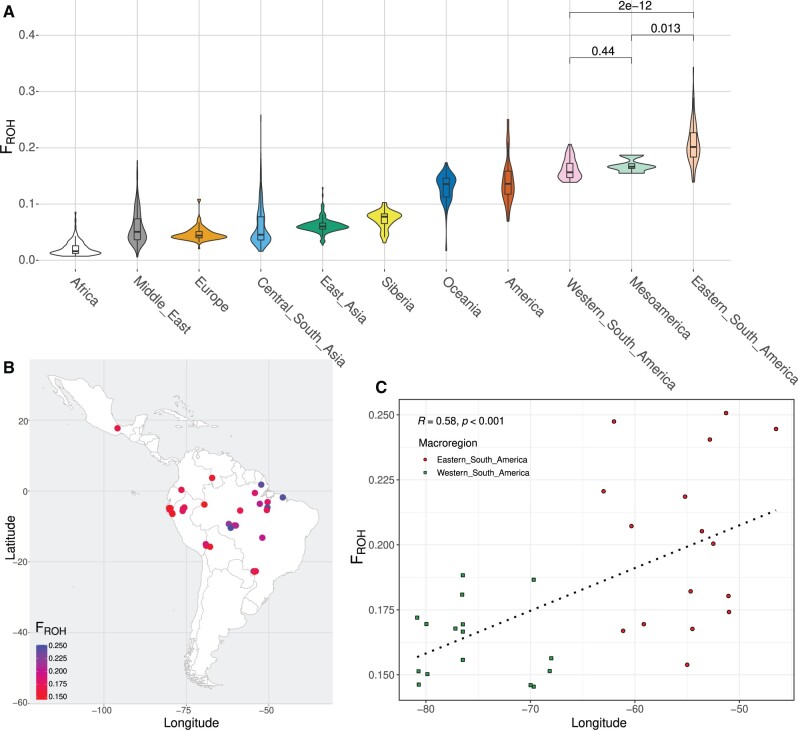
Distribution of inbreeding coefficient from ROH in Native Americans. (*A*) The distribution of *F*_ROH_ was obtained averaging the individual estimates from a combined set of the unadmixed Native Americans along with HGDP and SGDP databases (Africa, Middle East, Europe, Central South Asia, East Asia, Siberia, and America). The *P* values were obtained from a nonparametric Wilcoxon rank-sum test. (*B*) Population average estimates of *F*_ROH_ were plotted according to the corresponding geographic location. (*C*) Correlation of *F*_ROH_ values according to the longitude of each population. The dotted line was estimated by linear regression. The Spearman correlation coefficient and its corresponding *P*-value are also presented.

Finally, the eastern South Americans had significantly higher levels of the population inbreeding coefficient *F*_ROH_ (fig. 7 and supplementary fig. S18, Supplementary Material online), obtained from the runs of homozygosity (ROH), in comparison with that observed for the western South Americans, which means that the genetic diversity estimated for lowlander populations is significantly lower (*P* value = 2 × 10^−12^; fig. 7*A*). This suggests that populations close to the Amazonia were subjected, on average, to more extreme genetic drift processes, such as serial bottlenecks followed by subsequent founder events, which would have considerably reduced their genetic variability. This is therefore also consistent with the ASCEND and IBDNe analyses results, which point to more intense bottlenecks, on average, in eastern South America (fig. 6 and supplementary figs. S16 and S17, Supplementary Material online). When the longitude coordinates are considered, we observed that the farther east the population is located, the greater its *F*_ROH_ value was (Spearman correlation coefficient *r* = −0.58; *P* value < 0.001; fig. 7*C*), showing that the genetic variability increases according to proximity to the Andes region, which is consistent with the occurrence of serial population bottlenecks. Likewise, it indicates that Amazonia might be the region with the lowest genetic variability in the Americas, and thus, probably the lowest in the world. We also analyzed the distribution of IBD shared within populations and homozygosity by descent (HBD), which demonstrated a distinctive pattern between these regions, with ubiquitously lower IBD and HBD levels in the highlands, Pacific Coast, and Mesoamerica, and the opposite pattern in the eastern South American lowlands (supplementary fig. S19, Supplementary Material online), as expected.

## Conclusions

South American genetic variation is related to linguistic and environmental diversity, which is more pronounced in local contexts and within the same ethnolinguistic groups. We found at least four primary clusters of genetic similarity in the Amazonian and savanna regions, partially mirroring the main linguistic diversity. Moreover, no hard genetic division between the Andes and the Amazonia was noted. Furthermore, genetic variation and the homozygosity level were correlated with longitude, supporting an isolation-by-distance model coupled with the effect of serial population bottlenecks, possibly tracing back to an initial settlement from the Pacific coast, as also suggested by the population history models. We also described extensive ancient genetic interchange among the eastern lowland populations, with reduced allele sharing between the eastern and western Amazonian lowlanders, and genetic evidence of cultural exchanges in the precontact times, leading to language replacement. In addition, the present-day Native American diversity partially recapitulates ancient macroregional ancestries. Finally, demographic analyses indicated that western South Americans were less affected by the process of European colonization and showed that the population size of some South American and Mesoamerican groups varied greatly over the past 2,000–3,000 years, with extreme events of population growth and reduction.

## Materials and Methods

### Data Set Assembly

Here we used the data set from Castro e Silva et al. (2021), which combined new and publicly available data sets, genotyped with the Axiom Human Origins array (Affymetrix/Thermo Fisher) or whole-genome sequenced, as described below. The data set includes Brazilian populations from the Amazonian Rainforest, from Southwestern Brazil (near the Paraguayan border), the central Brazilian plateau, and also from the Brazilian Atlantic Coast (Patterson et al. 2012; Skoglund et al. 2015; Castro e Silva et al. 2020). To increase the scope of the study and to enable the examination of how the human genetic diversity is patterned across the Amazon-Andes divide and also more broadly on the American continent, populations from Mexico, Colombia, Ecuador, and Peru were also included (Lazaridis et al. 2014; Mallick et al. 2016; Barbieri et al. 2019). The data set was also merged with the 1240K_HO data set assembly (v42.4), to include publicly available data for other Native American groups and potential unadmixed individuals (with no or negligible signal of contributions from non-Native American sources) from other present-day populations from the American continent, as well as the publicly available ancient samples from the Americas. The complete data set contains 383 individuals from 50 different present-day indigenous ethnolinguistic groups (supplementary data set S1, Supplementary Material online), although it contains more than one set of samples for some of these groups (e.g., Quechua_Cusco, Quechua_Cusco2, Quechua_Cusco3, Quechua.DG, and Quechua_Puno), thus including a total of 58 populations. Please refer to supplementary data sets S1–S3, Supplementary Material online for more information on the test samples (i.e., Native Americans), on the reference panel of samples, and the ancient samples (i.e., aDNA ancient samples from the Americas and Siberia) used in this study, respectively.

### Data Curation

Before any analysis was performed, some genome-wide summary statistics were estimated for the complete data set. Considering a threshold of missingness rate per individual of 10% no sample was removed from the data set. Although an insignificant number of SNPs (74 SNPs) present a missingness rate above 5%, though not exceeding 7%, which were removed. The data set contains a proportion of 10.96% of rare alleles (SNPs with a minor allele frequency lower than 1%). Following this initial evaluation, the complete data set was pruned to remove markers with a pairwise correlation above 20% (*r*^2^ > 0.2 inside a sliding window of 50 kb size and step size of 10 kb) thus producing an LD-pruned data set. Next, a supervised clustering analysis was performed with ADMIXTURE (Alexander et al. 2009) on a subset of the LD-pruned data set (keeping all of the samples from the American continent, Sub-Saharan Africa, and western Europe), to assess whether the samples from Native American communities and other populations from the American continent have genetic contributions from non-Native American sources. Finally, the LD-pruned data set was also assessed for the presence of related individuals, for this we used PLINK 1.9 (Chang et al. 2015) pairwise IBD estimation method to obtain the proportion of shared IBD between all pairs of individuals (i.e., PI_HAT = P(IBD = 2) + 0.5 * P(IBD = 1)), these estimates were used as input to PRIMUS (Staples et al. 2013) to identify the maximum set of unrelated individuals, considering the first degree of relatedness (PI-HAT > 0.375) as the threshold. The complete data set could then be filtered to remove admixed (<99% inferred non-Native American ancestry) and related individuals (PI-HAT > 0.375). Thus allowing the selection of the subset of unadmixed individuals (with 150 individuals from 33 indigenous groups), the subset of unrelated individuals (with 312 individuals from 58 indigenous groups), and the subset unadmixed and unrelated individuals (with 87 individuals also distributed in 33 groups) (supplementary data set S1, Supplementary Material online).

### Global Ancestry and Population Structure

Initially, PCA was applied with SNPRelate R/Bioconductor (Zheng et al. 2012) to the LD-pruned subset of unadmixed and unrelated Native Americans and one additional PCA excluding outliers (Xavante, Xikrin, Karitiana, and Suruí), to examine the broad patterns of ancestry and genetic differentiation. Next, we applied ADMIXTURE (Alexander et al. 2009) to the unrelated and to the unadmixed-unrelated LD-pruned data sets to estimate the individual global ancestry components and to investigate the population structure and the patterns of shared ancestry of the South American indigenous groups. To do this, we executed one unsupervised ADMIXTURE analysis for each data set, with the number of putative genetic components K ranging from 2 to 10. To evaluate the estimated ancestry components we used the distribution of cross-validation errors and likelihoods (provided by ADMIXTURE) for each value of K, and we used PONG (Behr et al. 2016) to evaluate multimodality and plot the estimates. The proportions of individual ancestry components were also plotted on maps using the mean group values of these components and the geographical coordinates for each group, highlighting their linguistic affiliations. To do this some R packages were used as described below, “ggmap” to obtain the map, data for the main rivers of the American continent was provided by “mapdata” (wider blue lines), and data for the main rivers of Brazil (thin blue lines; which were not added to all map figures to avoid overplotting) was obtained from the website of the Laboratório de Pesquisas em Geografia Física (LAPEGE; http://www.uel.br/laboratorios/lapege/pages/base-de-dados-br.php; last accessed October 27, 2020), “scatterpie” to plot the pie charts on the map, and “ggrepel” to create labels for each group.

### Patterns of Shared Ancestry

As a first step, we analyzed how identity by descent (IBD) blocks are shared between the entire set of pairs of Native American groups and how these connections were distributed along the geographical space, following the same approach used by Barbieri et al. (2019). To do so, the subset of Native American groups’ data was filtered to remove markers, and samples with more than 5% of missingness, monomorphic SNPs were also pruned. Then, this data set was phased with BEAGLE v.5.1 (Browning et al. 2018a), the IBD segments were identified with Refined IBD (Browning and Browning 2013) and a Local Ancestry Inference was performed with RFMix (Maples et al. 2013), with a window size of 0.2 cM, with 5 as the minimum number of reference haplotypes per tree node, and the unadmixed Native Americans, Sub-Saharan Africans, and western Europeans, as the reference panel of populations. Next, the short gaps in the IBD segments were removed with the program merge-ibd-segments.17Jan20.102.jar, using default parameters (i.e., 0.6 as the maximum gap length and 1 as the maximum number of discordant homozygotes), then the IBD segments were classified and separated into different subsets according to their local ancestry.

We only analyzed IBD segments of Native American ancestry, identified in the maximum unrelated subset of individuals, and to secure reliable results we selected blocks with more than 2 cM and with an estimated LOD score above 3 (Browning and Browning 2013), furthermore only pairs of populations with more than 5 cM of IBD on average were considered as population pairwise sharing. The IBD blocks were then classified into different length categories essentially corresponding to distinct approximate time periods (Baharian et al. 2016; Harris et al. 2018). According to Baharian et al., the past generation time when an IBD segment was formed can be inferred from its length in morgans using the approximation: E(generations ago) ≅ 3/2I; I = IBD segment length in morgans. In our case, we have selected three approximate periods: 1) Pre-Columbian period ∼< 1500 CE (segments <= 8.4 cM); 2) colonial period ∼ 1500–1850 CE (8.4 cM < segments <= 28 cM); and recent period ∼ 1850–present CE (28 cM > segments). Where 8.4 cM corresponds to 500 years ago (17.85 generations ago * 28 years per generation), and 28 cM corresponds to 150 years ago (5.35 generations ago * 28 years per generation). The average number of IBD segments and the average length of shared IBD were then calculated for each length category independently, with in-house R scripts, by dividing these averages, one at a time, by the product of the sample sizes of the 2 populations being compared. The map of pairwise connections was produced with the “ggmap” R package.

Next, to assess the patterns of allele sharing between indigenous groups and to test prior hypotheses regarding the formation of some specific groups, we first estimated the Outgroup *F*_3_ (*Y*, *Z*; Mbuti), as well as the formal test *F*_4_ (Mbuti, Test; *Y*, *Z*). Both statistics (*F*_3_ and *F*_4_) were calculated for every pair of *Y* and *Z* indigenous groups, and “Test” was iterated over every single indigenous group or individual in the *F*_4_ statistics computation. Additionally, a matrix of Outgroup *F*_3_ (*Y*, *Z*; Mbuti) calculated for all *Y* and *Z* pairs of groups and pairs of individuals, was converted to genetic distances (Genetic distance = 1 − Outgroup *F*_3_ estimate). A MDS analysis was then applied to the matrix of pairwise genetic distances with the “stats” R package.

### Patterns of Genetic Continuity

To shed light on the local patterns of genetic continuity in the South American continent, first, we wanted to investigate the relative patterns of excess affinity between ancient individuals and each pair of present-day Native American groups, by computing *F*_4_ (Mbuti, *X*; *Y*, *Z*), where *X* is any ancient sample, and *Y* and *Z* are any pair of present-day Native American groups. First, we filtered the highly significant tests (*Z* > 4) and then the selected set was summarized as the number of significant tests, grouping by pairs of *X* ancient individuals and *Z* present-day groups, and summing over all the *Y* present-day groups. We also estimated the pairwise genetic distance (1 − Outgroup *F*_3_ estimate) matrix between all present-day (Native Americans) and ancient (from the American continent and Siberia) individuals and applied an MDS to this matrix using the “stats” R package.

### Genetic Diversity and Geography

Furthermore, we wanted to investigate if and how much the genetic diversity of indigenous populations was influenced by their geographic distances. We used the 1 − Outgroup *F*_3_ as the measure of genetic distance and the geographical distances were calculated based on the coordinates of every pair of groups as great circle distances with the R package “geosphere.” We then applied a linear regression model (R “stats” package) to the matrices of genetic and geographical distance of all 87 indigenous individuals from the unadmixed-unrelated data set. Finally, we looked at the relationship between continental ancestry components (i.e., Native American, African, and European) and Latitude/Longitude, and also between the estimated principal components and the latter, by fitting linear regression models.

### Population Histories and Admixture Events in Present-Day Indigenous Groups

First, we aimed to produce an outline of the population history of the Native American groups here represented, therefore providing subsidies, along with other lines of evidence from archeology and linguistics, for a framework to model how these groups relate to each other and for how their population history unfolded. This was initially explored with Treemix program (Pickrell and Pritchard 2012), which uses an unsupervised method for estimating the maximum likelihood tree based on population pairwise allelic covariances and allows for putative gene flow events to be adjusted between branches of the tree.

Second, we used the qpGraph (v.6450) from Admixtools (Patterson et al. 2012) to model the population history of the present-day indigenous groups, by compiling several F statistics to infer the best fit between the genetic variation observed and the model. To do so, we computed the models with the unrelated and unadmixed set of Native American individuals, using only groups/individuals that presented a coverage of at least 200k SNPs. The default settings for qpGraph were used, except for the parameters “outpop: NULL” and the “all SNPs: YES.” A scaffold tree composed of groups representing the major possible contributions for the tested groups (Mesoamericans; Pacific Coast; Andes; Amazonian; Tupí-speaking groups; western Amazonia: Shawi, Muniche; and Jê-speaking groups) was obtained by progressively including the most divergent groups and selecting the best-fitted models (i.e., lowest maximum |*Z*|) as candidates to represent the population history.

Next, we modeled the test groups in all possible placements along the scaffold branches, both as one-way models assuming a single origin of the tested group or as two-way models assuming a mixed origin. When both models (single and admixed origin) presented a good fit, we gave preference to the single-origin model (one-way), as it is the more parsimonious scenario. However, if among the multiple models there was no one-way with a good fit, we used the criteria of the lowest maximum Z score among all two-way models and the number of outliers as a proxy to decide which is the best candidate model to explain the genetic patterns observed. In search of the best fit, we tested one group at a time (Kokama_Peru, Kokama_Colombia, Kaiowá, Ñandeva, and Mbyá), placing the test group in all terminal positions.

### Demographic Inferences

We then used the ASCEND software (Tournebize et al. 2020) to infer the age and intensity of the bottleneck events on the Native American groups. First, we selected the Native Americans from the complete data set, then we pruned markers and samples with more than 5% of missing data (no samples were removed due to this criterion). Next, we selected groups with more than 5 unrelated samples (keeping samples with evidence of admixture with other continental ancestries), to ensure the minimum required sample size (*N* ≥ 5) and to avoid the confounding effect of consanguinity. ASCEND infers the age and intensity of founder events (or bottlenecks in this case) as parameters of a model based on the empirical curve of exponential decay of allele sharing correlation between pairs of individuals inside the same population as a function of the genetic distance. The cross-population correlation with an outgroup is subtracted from this intra-population correlation to exclude the effect of ancestral allele sharing. A random sample of 15 individuals from the complete data set was used as the outgroup population as in Tournebize et al. (2020).

We also analyzed the IBD segments identified as described previously in the section “Patterns of Shared Ancestry”, first by selecting segments shared between individuals from the same population (i.e., intra-population IBD) and present in genomic regions of Native American ancestry as inferred with RFMix (Maples et al. 2013), and second by selecting segments shared within the same individual (i.e., HBD). Then, these segments were binned into five length categories (1–2, 2–4, 4–8, 8–16, >16), which are informative about events that happened in different time frames (longer segments were formed more recently, and vice-versa). Then the average length and number of HBD were calculated for major groups as well as for populations, and finally, the average length of IBD shared in the intra-population level was also estimated. The data were grouped by the segment length categories and the averages were obtained for each of them.

Additionally, we used IBDNe (Browning et al. 2018b) to estimate *N*_e_ history also based on the inferred IBD segments (section “Patterns of Shared Ancestry”), but first, we selected those located inside the blocks of Native American ancestry identified through a Local Ancestry Inference conducted with RFMix (Maples et al. 2013). The IBDNe was then applied to the IBD segments of Native American ancestry to infer the ancestry-specific *N*_e_ history, using the default parameters, except for the parameter “filtersamples=false,” which was used due to the small sample sizes per group, and for this reason, we did not exclude the related samples from the analysis and therefore the estimates obtained for the most recent generations are expected to be particularly biased by the presence of relatedness between samples. To assess this bias, we also performed a second set of analyses in which we used the parameter “filtersamples=true,” hence removing related samples, and it is presented in the Supplementary Material.

Finally, we combined the unadmixed set of Native Americans, with a data set containing information from 952 individuals from worldwide populations from the Human Genome Diversity Project (HGDP) (Bergström et al. 2020) and Simons Genome Diversity Project (SGDP) (Mallick et al. 2016), resulting in a merger of 1,102 individuals. Then a quality control was performed by removing: 1) autosomal triallelic markers, 2) SNPs within the 2 Mb of the extremities of all chromosome arms, 3) loci with extreme deviations from Hardy–Weinberg proportions (*P* value ≤ 10^−8^), and 4) SNPs with more than 10% of missing values. The resulting set of markers is composed of 251,940 autosomal SNPs.

Next, the ROH identification was performed with PLINK v1.9 (Chang et al. 2015), using a sliding window of 50 SNPs, with a maximum of five missing calls and one heterozygous genotype per window, a proportion of 5% of overlapping windows in a homozygous segment, with at least one SNP each 50 kb, a maximum gap of 100 kb between consecutive SNPs, and a minimum ROH length of 500 kb. The ROH was used to estimate the individual inbreeding coefficient from ROH, as proposed by McQuillan et al. (2008), in which the estimate corresponds to the genomic proportion composed by ROH, that is, the total ROH length divided by the total genomic region covered by the SNPs. Then, population *F*_ROH_ averages were estimated from the individual values. Last, the *F*_ROH_ estimates were compared with the geographic position of these groups, by assessing the correlation between the *F*_ROH_ estimates for each group with their Longitude and Latitude.

## Supplementary Material

Supplementary data are available at *Molecular Biology and Evolution* online.

## Supplementary Material

msab339_Supplementary_Data
