# Supplementary material for: Population Histories and Genomic Diversity of South American Natives
Source: Mol Biol Evol. 2021 Dec 8;39(1):msab339. doi: 10.1093/molbev/msab339 (PMC8789086; doi:10.1093/molbev/msab339)
Supplement: msab339_Supplementary_Data [file msab339_supplementary_data.zip › Supp_Material_CS_rev.pdf]

Supplementary Information for:

Population histories and genomic diversity of South American natives

Marcos Araújo Castro e Silva[a], Tiago Ferraz[a], Cainã M. Couto-Silva, Renan B. Lemes[a], Kelly Nunes[a], David Comas[b] and Tábita Hünemeier[a]

E-mail: hunemeier@usp.br

[a]*Departamento de Genética e Biologia Evolutiva, Instituto de Biociências, Universidade de São Paulo, São Paulo, SP, Brazil;* [c]*Institut de Biologia Evolutiva, Departament de Ciències Experimentals i de la Salut, Universitat Pompeu Fabra, 08003 Barcelona, Spain*

This PDF file includes:

Supplementary Table S1

Supplementary Figures S1 to S19

Legends for Datasets S1 to S6

Other supplementary materials for this manuscript include the following:

Datasets S1 to S6

Supplementary Tables

Table S1 - Influence of ethnolinguistic diversity on genetic variation. An AMOVA (Excoffier et al. 1992) was applied using the ‘poppr’ R package (Kamvar et al. 2014) to the subset of unadmixed and unrelated samples, keeping only SNPs with no missing data and pairwise correlation below 1% (selecting 10,236 SNPs).

	Variation including within-individual level (%)[1]	p-value[2]	Variation (%)
Variations Between Major Group	1.0811	0.004*	16.2223
Variations Between Group Within Major Group	2.4328	0.001*	36.5069
Variations Between samples Within Group	3.1501	0.1480	47.2708
Variations Within individual	93.3360	0.002*	
Total variations	100.0000		100.0000

*Statistically significant values; [1]Genotypes are separated into haplotypes and used to calculate within-individual variation (equivalent to the default setting of the Arlequin program); [2]Significance estimated with the randomization test from ‘ade4’ package.

Supplementary Figures

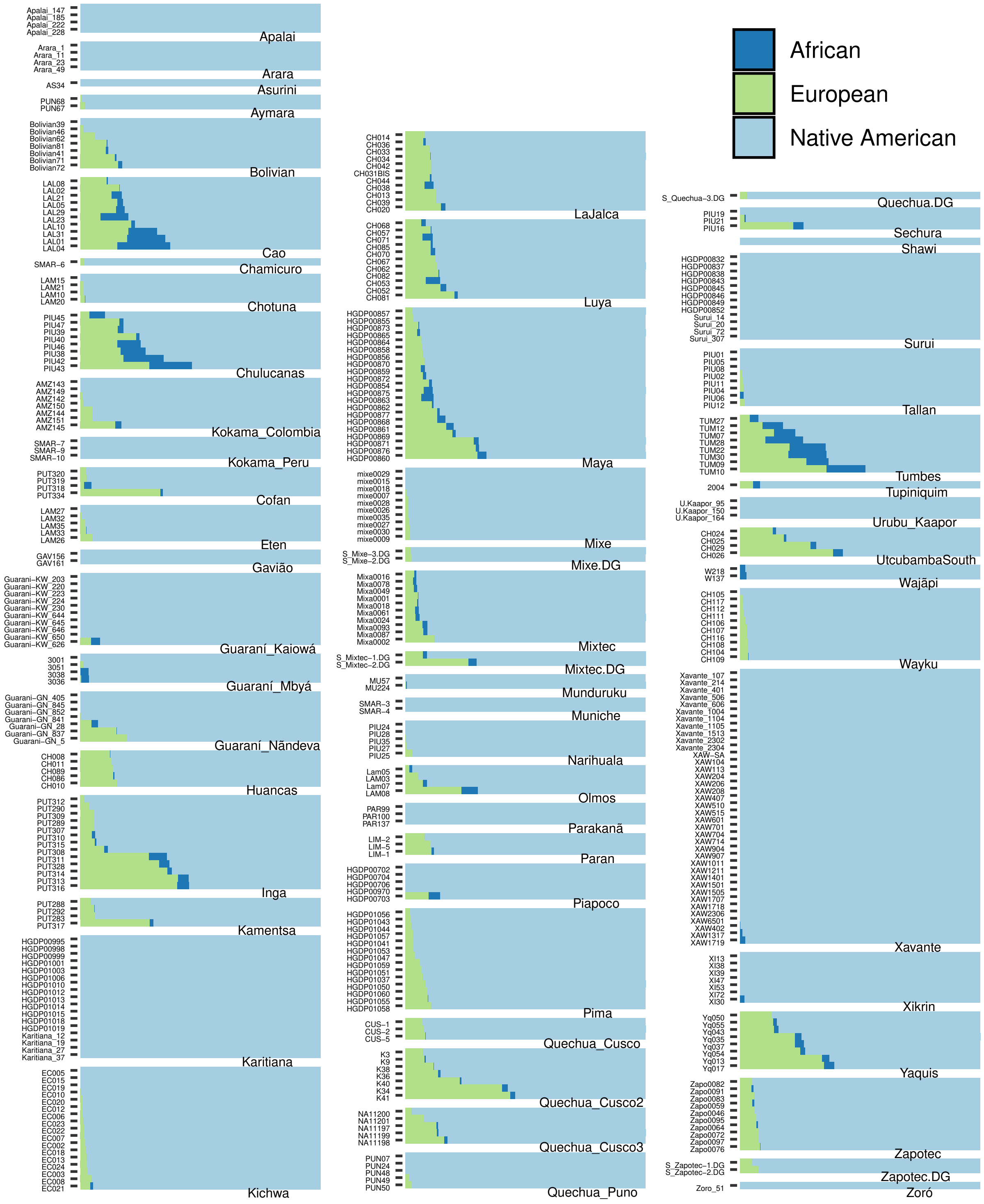

Figure S1 - Non-Native American admixture profile of American indigenous groups. An unsupervised ADMIXTURE (Alexander et al. 2009) analysis on the subset of Native Americans with K = 3 was performed in order to estimate their proportions of African and European admixture. The African populations used as proxies of the parentals were Bantu from Kenya, Bantu from South Africa, Biaka, Mandenka, Mbuti, and Yoruba from HGDP, additionally, the European populations used as parentals were Basque, French, Italian, Orcadian, Sardinian, Tuscan from HGDP, and Basque, Southern Italian, Sicilian, Spanish, Northern Spanish from Lazaridis et al. (Lazaridis et al. 2014; Lazaridis et al. 2016). The three panels exhibit the individual proportions of African (dark blue), European (green), and Native American (light blue) ancestries, individual labels are

placed on the left side of each bar and group labels are placed at the bottom right of each group. A total of 150 unadmixed individuals can be identified and they are listed on the Dataset S1.

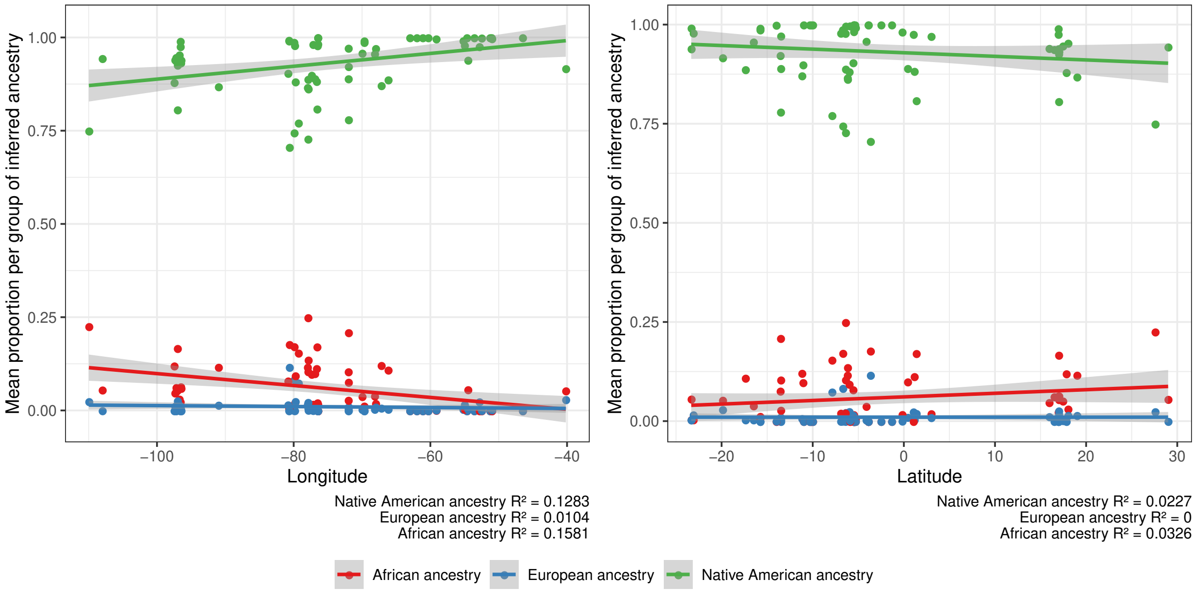

Figure S2 - Admixture proportions components in relation to geography. Here we use the continental ancestry components in the analysis presented in Figure S1, to test if the ancestry proportions were correlated with the geographic position (longitude and latitude) of each group. The two panels show the mean proportion of each inferred ancestry (see bottom legend) for each group in the form of color-coded points as functions of their longitude (left) and latitude (right), additionally, a linear regression model is fitted for each ancestry component along with their 95% confidence interval presented as shaded areas. The coefficients of determination (R^2) of each model are shown at the bottom right of both panels.

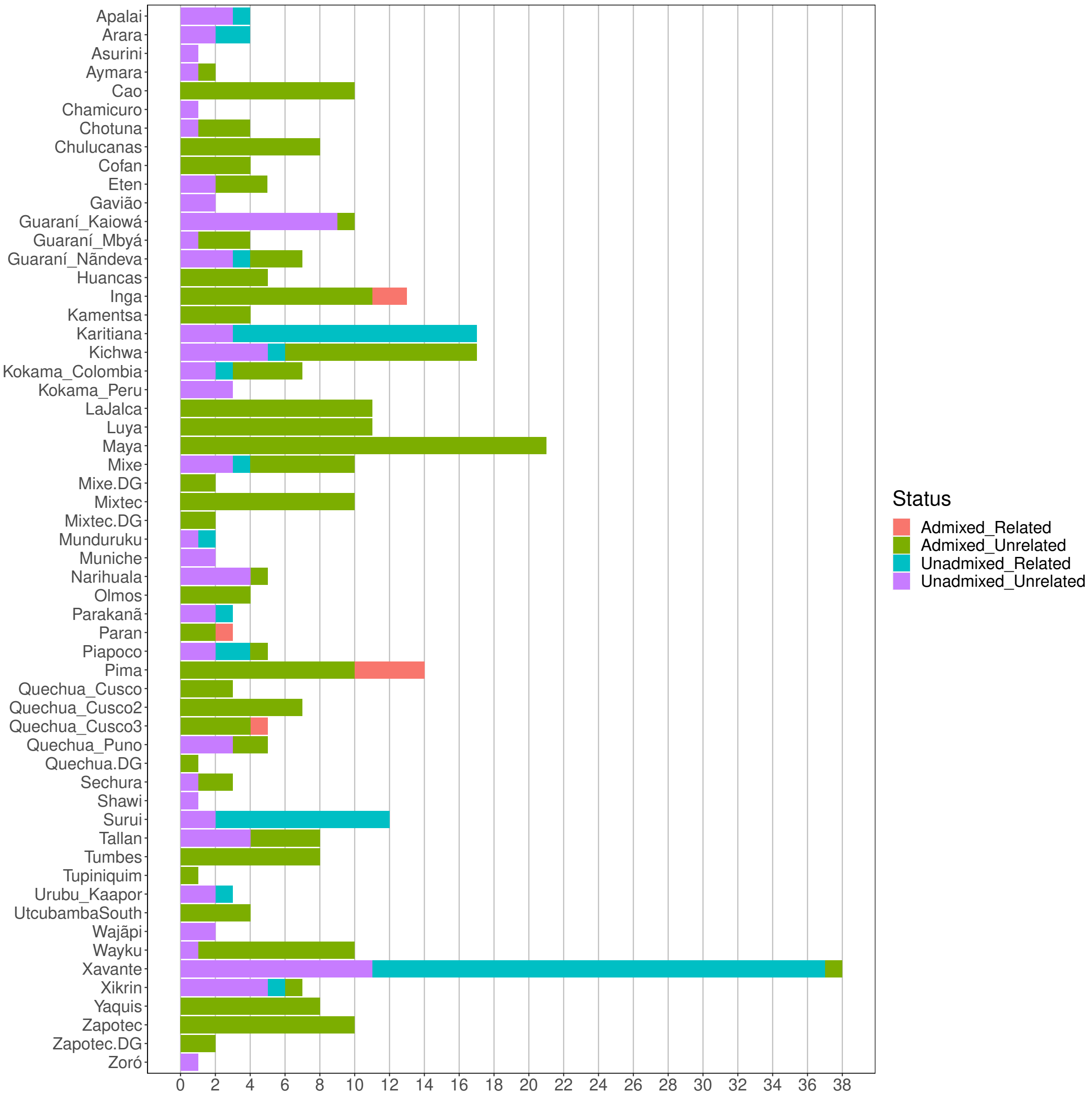

Figure S3 - Assessing non-Native American admixture and relatedness in the set of Native American groups. Individuals are classified into the categories unadmixed and unrelated, respectively when they present more than 99% of inferred Native American ancestry in ADMIXTURE (Alexander et al. 2009) analysis and are selected in the maximum unrelated (or independent) set of individuals with a PI-HAT < 0.375 (1st-degree relatedness) with PRIMUS (Staples et al. 2013). Wajãpi individuals were included despite having a non-negligible contribution from non-Native American ancestors (~ 3%; see Figure S1 and Dataset S1).

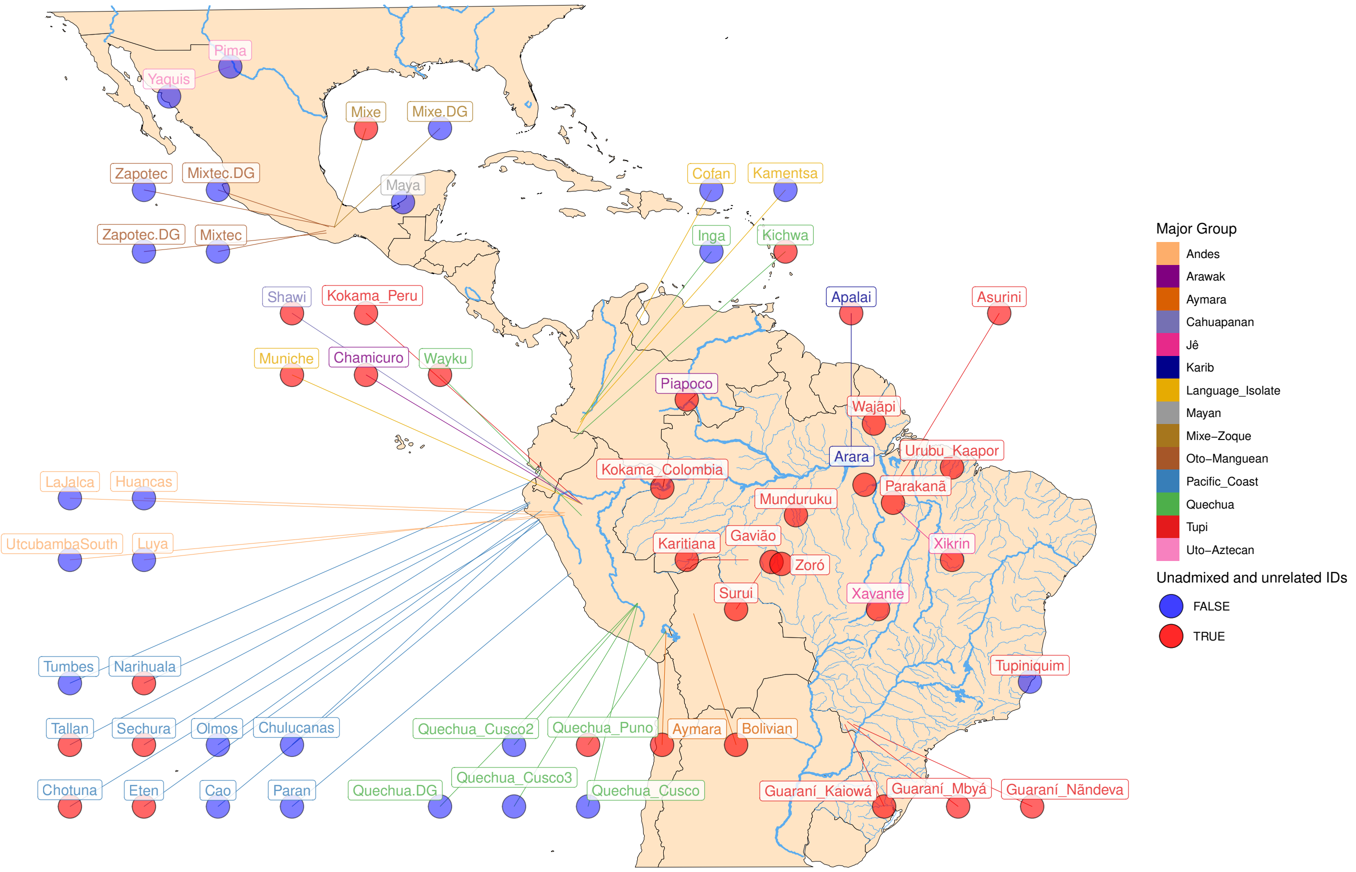

Figure S4 - Map of indigenous groups from the American continent. Labels and circles indicate the group names and their approximate location. Affiliation to the major groups (Table 1) used throughout this paper is color-coded in the labels and indicated on the legend at the right side of the plot. Finally, if a given group contains at least one individual unadmixed and unrelated to any other individual in the dataset, the circle is colored red and when this is not the case, blue.

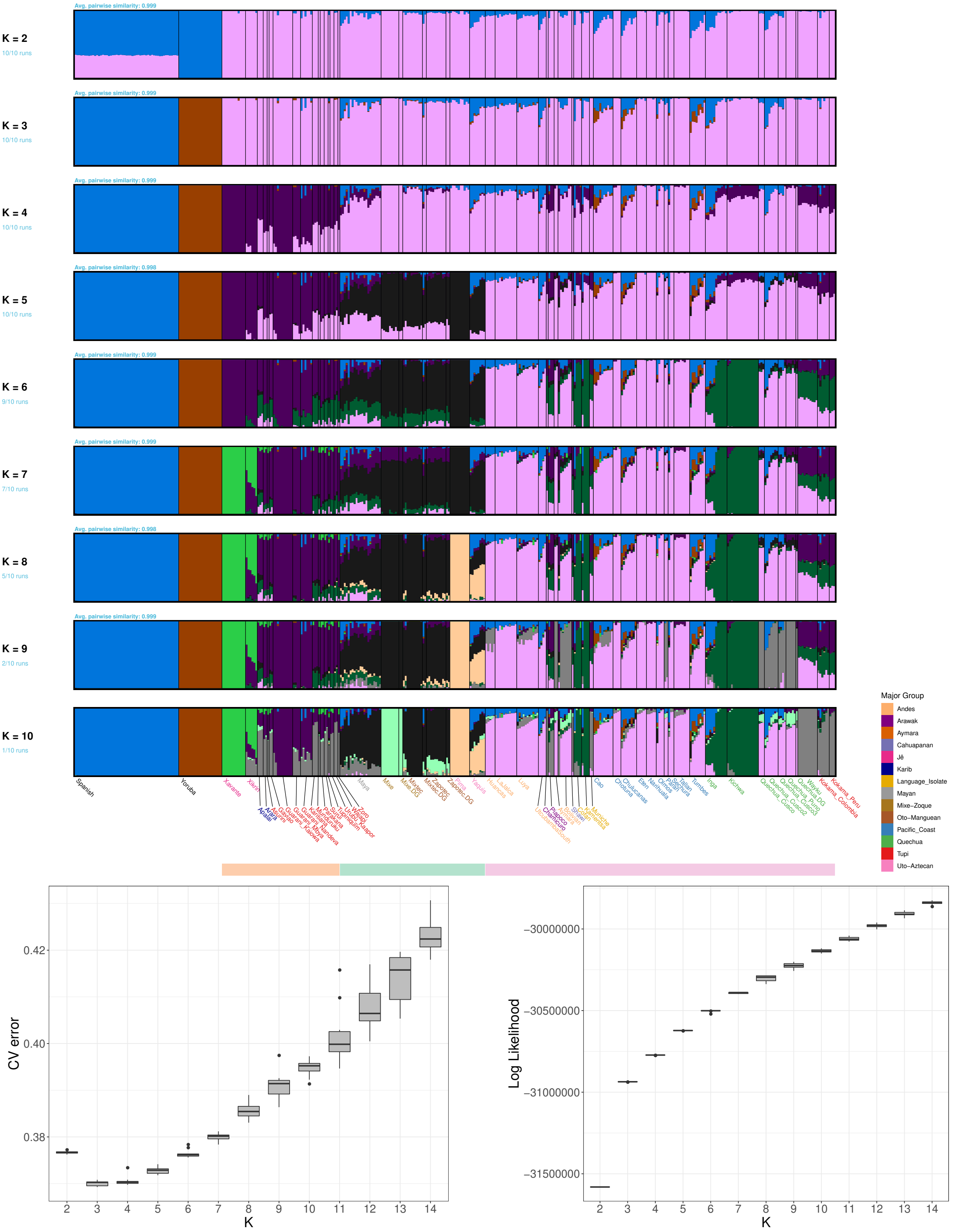

Figure S5 - Genetic structure and European and African admixture in Native Americans. Individual values of the putative ancestry components were estimated with an unsupervised ADMIXTURE (Alexander et al. 2009) analysis of the complete set of Native Americans with K values from 2 to 10 (Top panels) and the bar plots of the estimates were produced with PONG (Behr et al. 2016). The number of putative ancestry components tested increases from top to bottom, group labels are given at the bottom of the last barplot and they are color-coded to indicate their affiliation to major groups, as shown in the legend at the bottom right. The three main continental regions are indicated by the colored bar at the bottom: Mesoamerica in light green, western South America in pink, and eastern South American in beige. The cross-validation error (bottom left) and likelihood (bottom right) of each iteration of the algorithm were also estimated by ADMIXTURE.

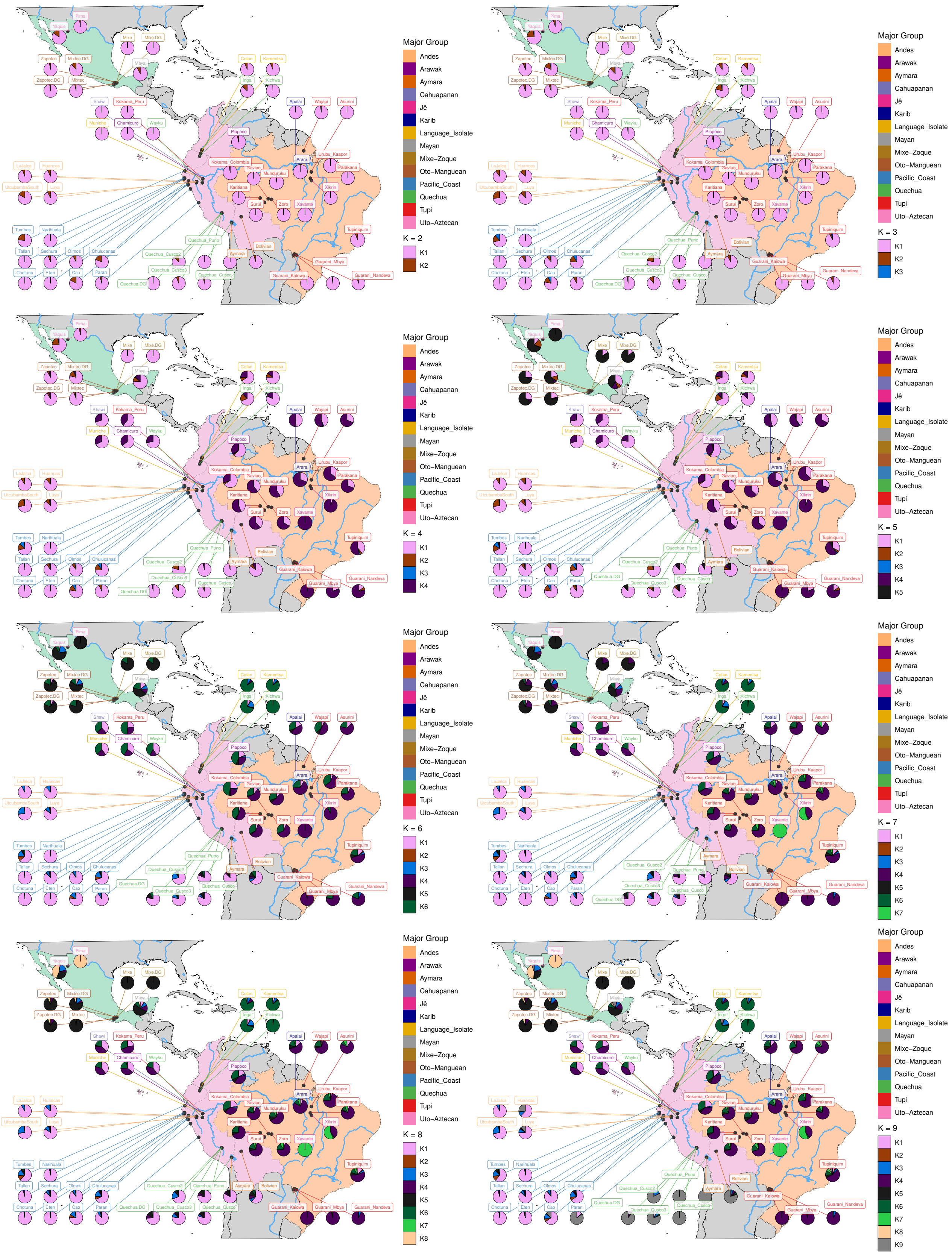

Figure S6 - Mean ancestry components of the complete set of Native American groups. Population mean values of the putative ancestry components were estimated with an unsupervised ADMIXTURE analysis with K values from 2 to 10 of the complete set of Native Americans (same estimates used in Figure S4) and plotted as pie charts a map of Central and South America, based on the approximate sampling location of each group. The linguistic affiliations as well as the putative ancestry components (K) are color-coded as indicated in the legend at the right.

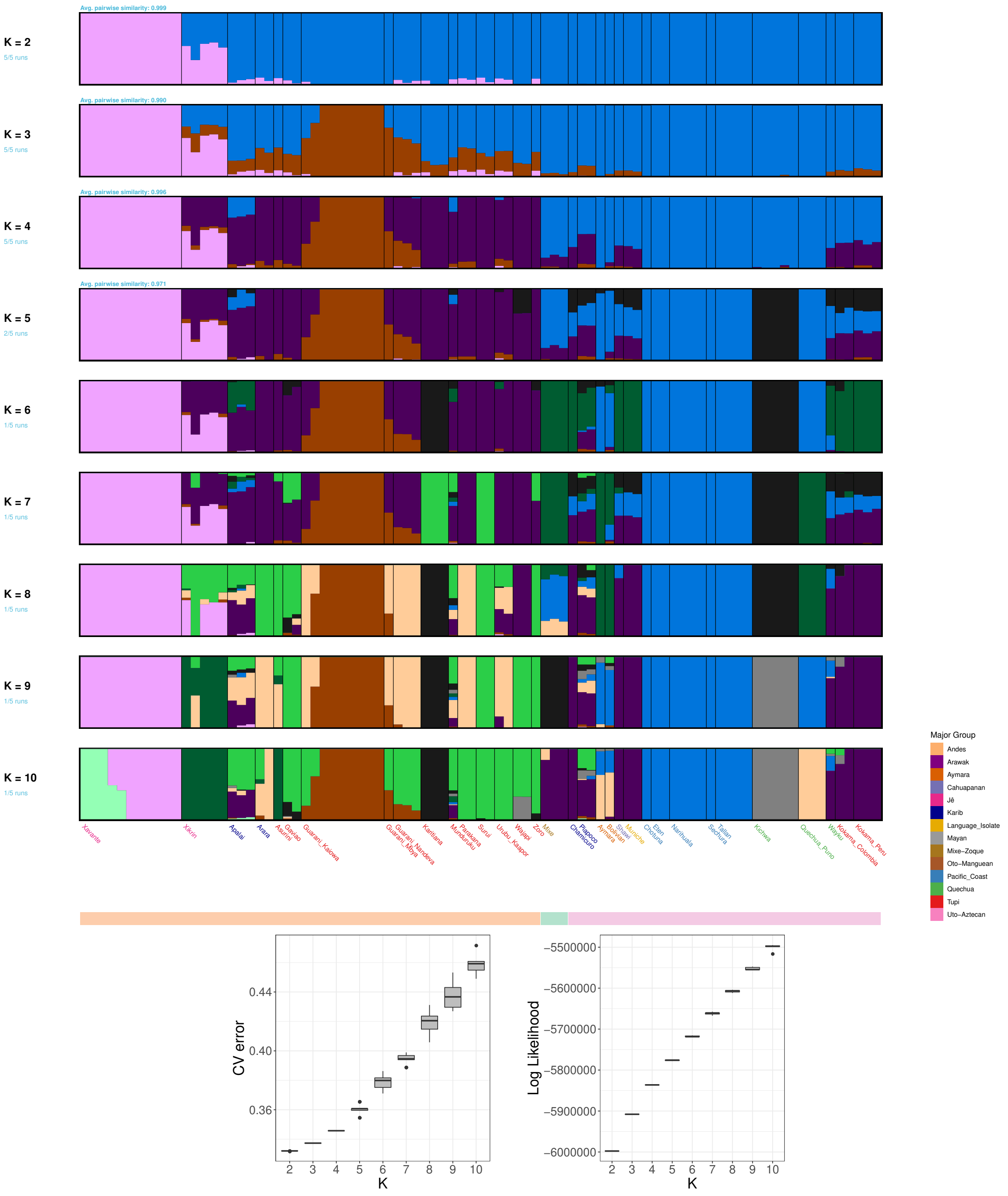

Figure S7 - Genetic structure and patterns of shared ancestry in unadmixed and unrelated Native Americans. Individual values of the putative ancestry components were estimated with an unsupervised ADMIXTURE (Alexander et al. 2009) analysis of the subset of unadmixed and unrelated Native Americans with K values from 2 to 10 (Top panels) and the bar plots of the estimates were produced with PONG (Behr et al. 2016). The number of putative ancestry components tested increases from top to bottom, group labels are given at the bottom of the last barplot and they are color-coded to indicate their affiliation to major groups, as shown in the legend at the bottom right. The three main continental regions are indicated by the colored bar at the bottom: Mesoamerica in light green, western South America in pink, and eastern South American in beige. The cross-validation error (bottom left) and likelihood (bottom right) of each iteration of the algorithm were also estimated by ADMIXTURE.

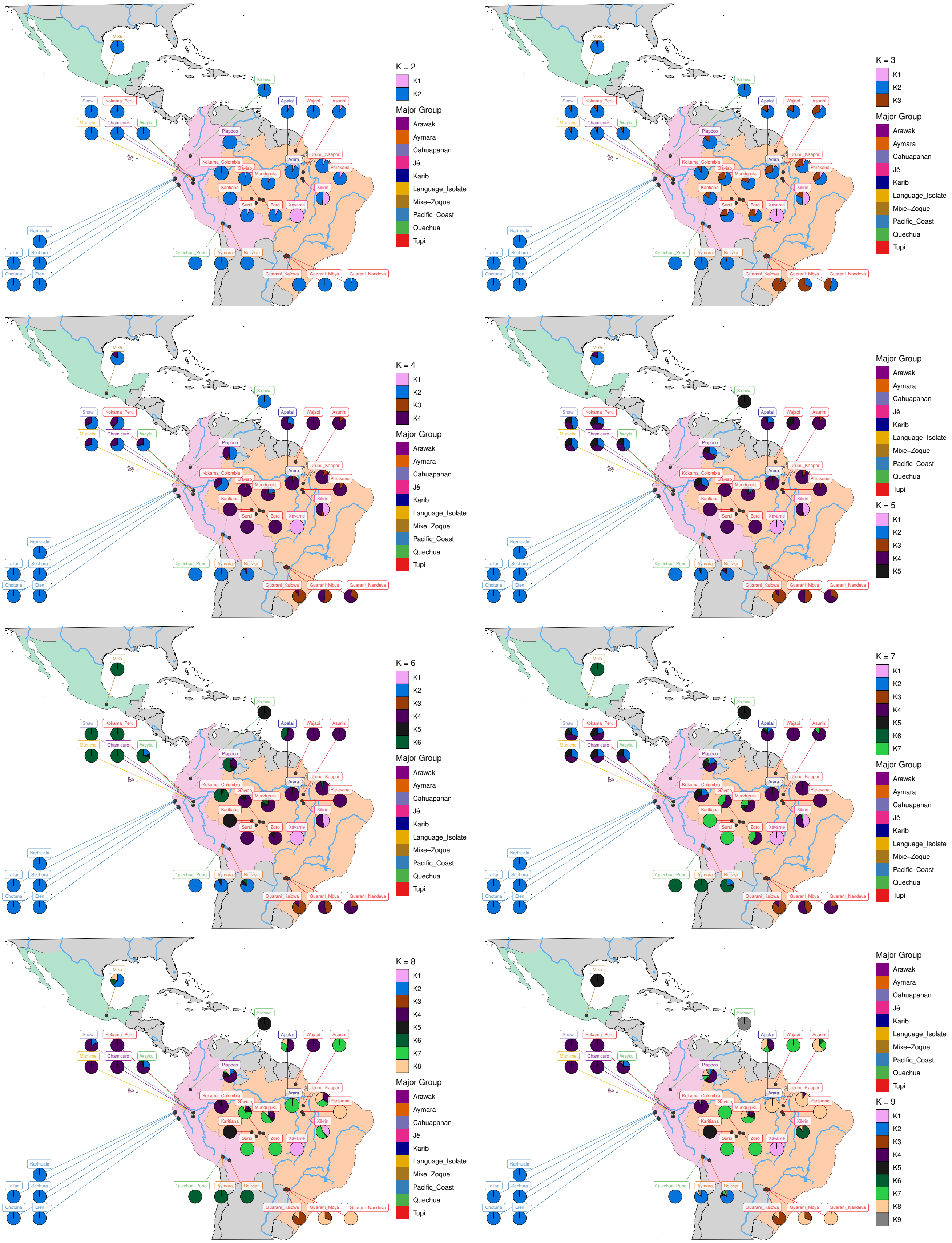

Figure S8 - Mean ancestry components of the subset of unadmixed and unrelated Native American groups. Population mean values of the putative ancestry components were estimated with an unsupervised ADMIXTURE analysis with K values from 2 to 10 of the subset of unadmixed and unrelated Native Americans (same estimates used in Figure S5) and plotted as pie charts a map of Central and South America, based on the approximate sampling location of each group. The linguistic affiliations as well as the ancestry components (K), are color-coded as indicated in the legend at the right.

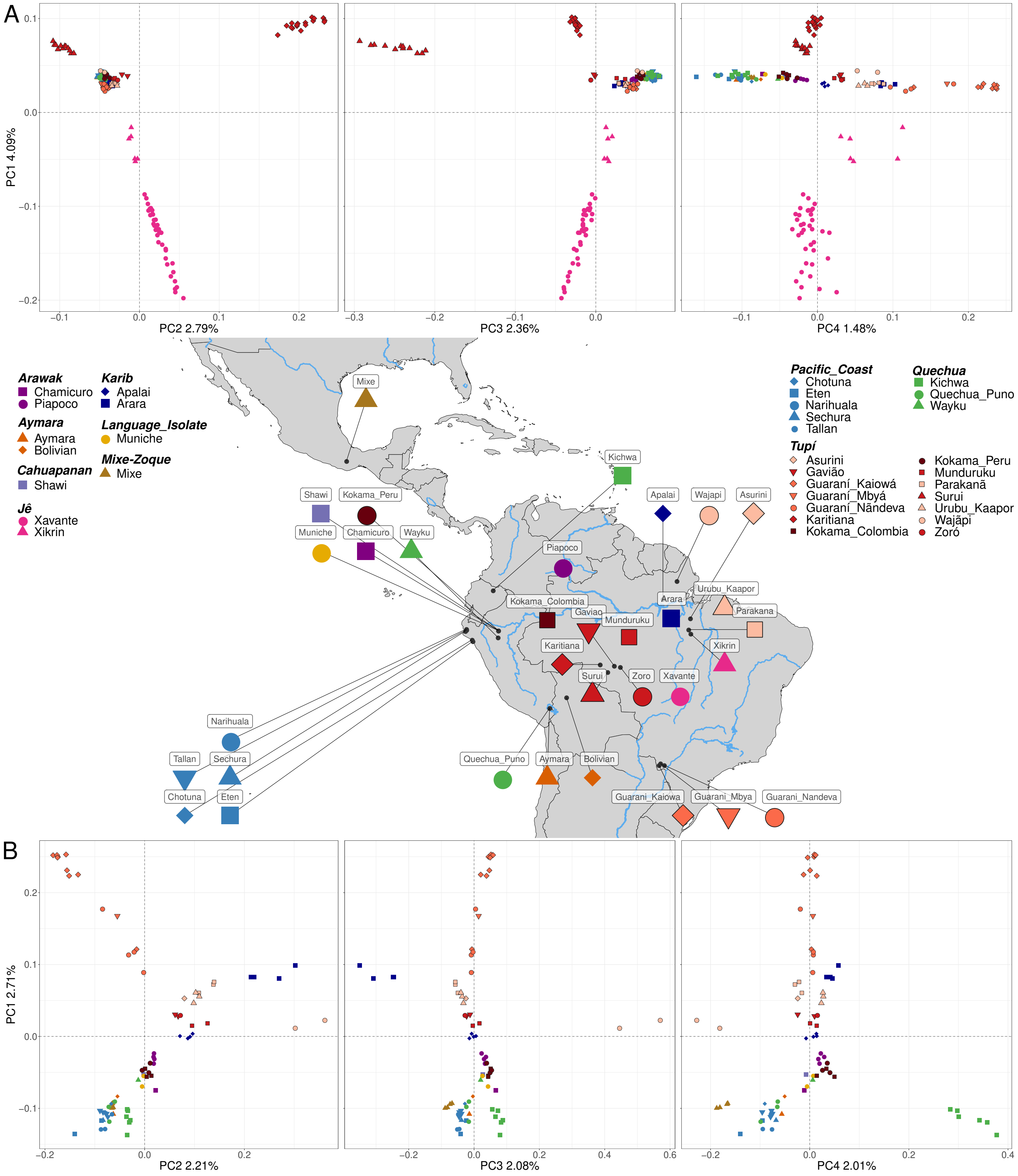

Figure S9 - Broad patterns of shared ancestry among Native Americans. A PCA was applied to **(A)** the LD-pruned set of unadmixed and unrelated Native Americans and to **(B)** a subset excluding the most divergent groups, namely: Xavante, Xikrin, Karitiana, and Suruí. The plot exhibits the combinations of the first PC (x-axis) with the second to fourth PCs (y-axis), from left to right: PC1 and PC2; PC1 and PC3; PC1 and PC4. The groups and major groups' affiliations (Table 1) are coded as shapes and colors, respectively, as indicated in the legend at the center of the plot.

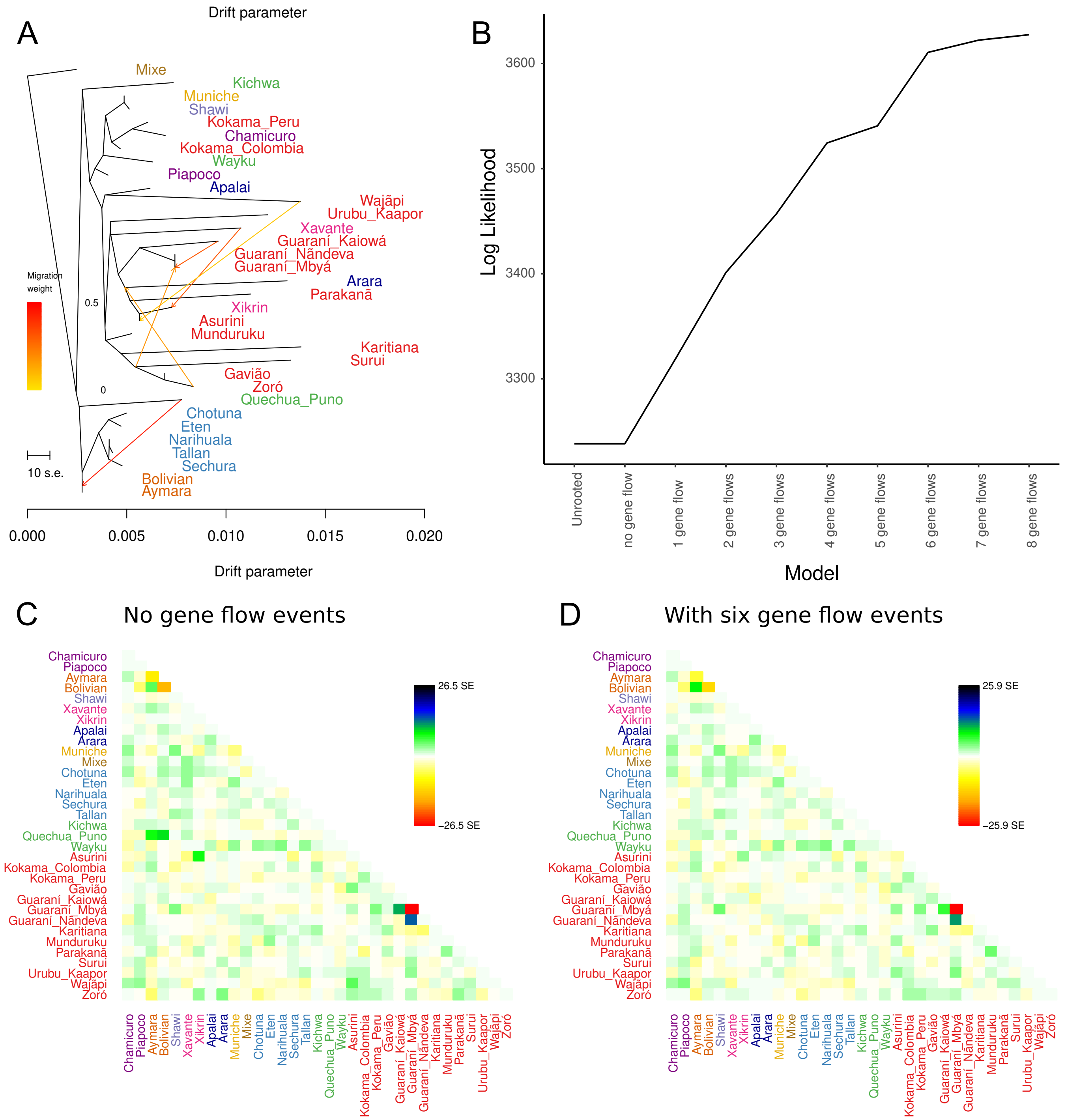

Figure S10 - Maximum likelihood tree modeling. Using Treemix (Pickrell and Pritchard 2012) Maximum Likelihood (ML) trees were estimated and gene flow events were modeled between poorly fitted branches. **(A)** ML tree with six gene flow events. **(B)** Plot showing the likelihood (y-axis) for each model (x-axis). **(C)** Residue matrix for the no gene flow model. **(D)** Residue matrix for the six gene flow events model.

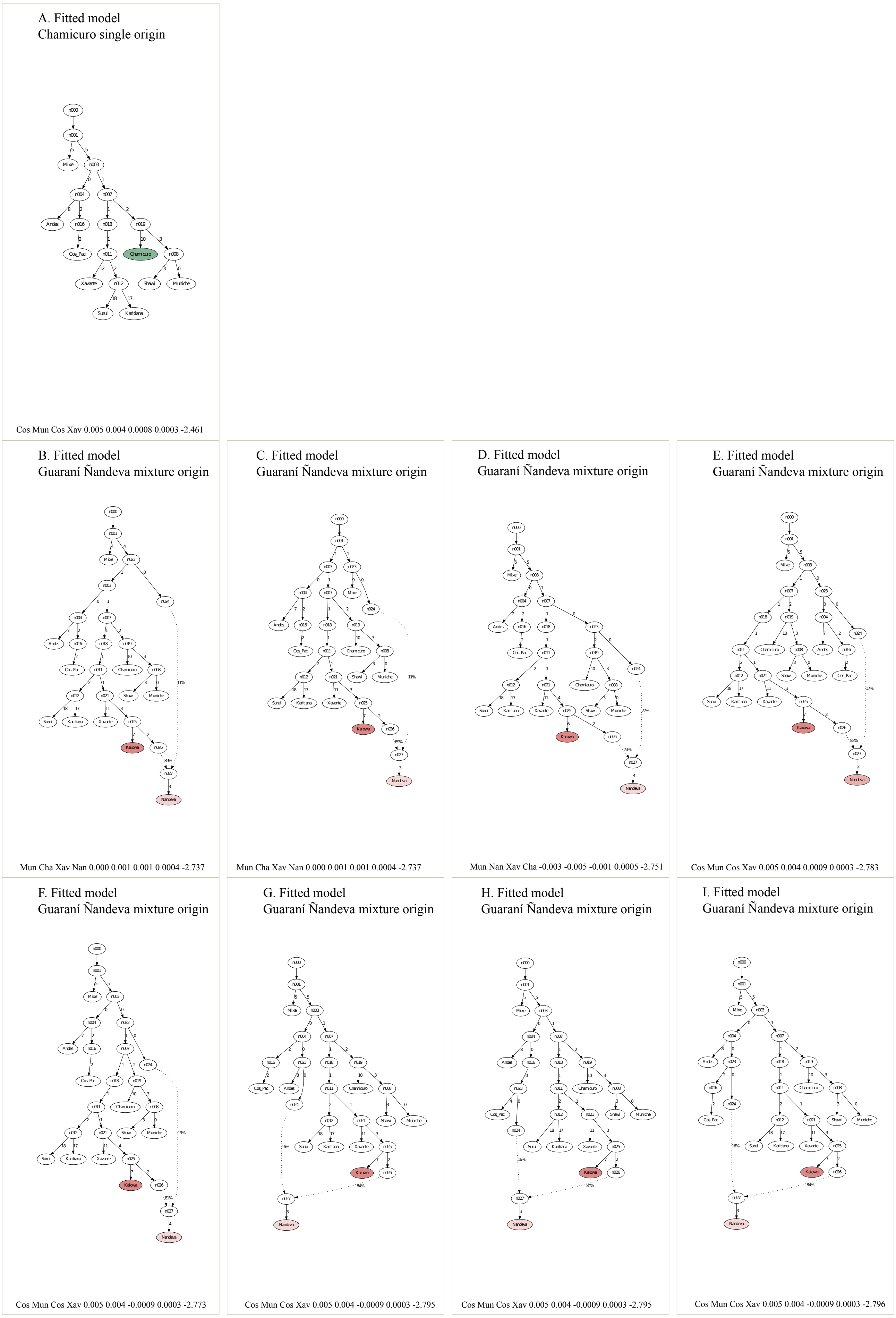

Figure S11 - Complementary set of admixture graph models. (A) Graphic representation of the best-fitted model for a single origin of Chamicuro - Arawak speakers - this model was used as a scaffold tree to build up all

the other models. **(B-I)** Models with a good fit to the data for a mixture origin of the Guaraní Ñandeva. Note that **B** is the same model presented in Figure 4C.

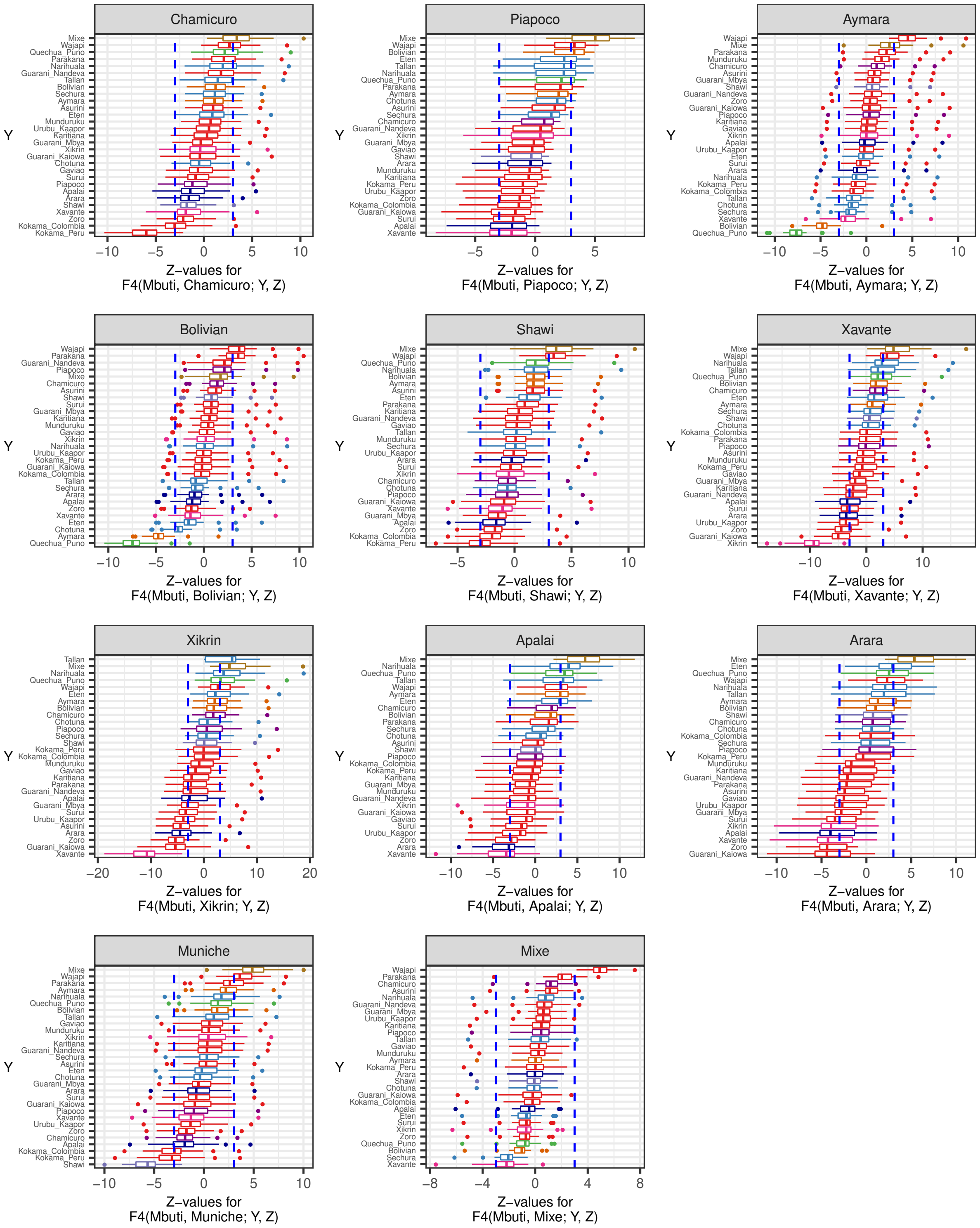

Figure S12 - Genetic affinities among present-day indigenous groups. To examine the patterns of allele sharing we estimated F_4(Mbuti, X; Y, Z) for every combination of X, Y, and Z present-day Native American groups. Each panel shows the combinations of X (top stripe) and Z (y-axis) test groups, and the Z-values (x-axis) obtained by the comparison with every Z test group are presented in the form of boxplots. Panels ordered by major groups (Table 1). The complete set of statistics is presented in Dataset S6A. (Start)

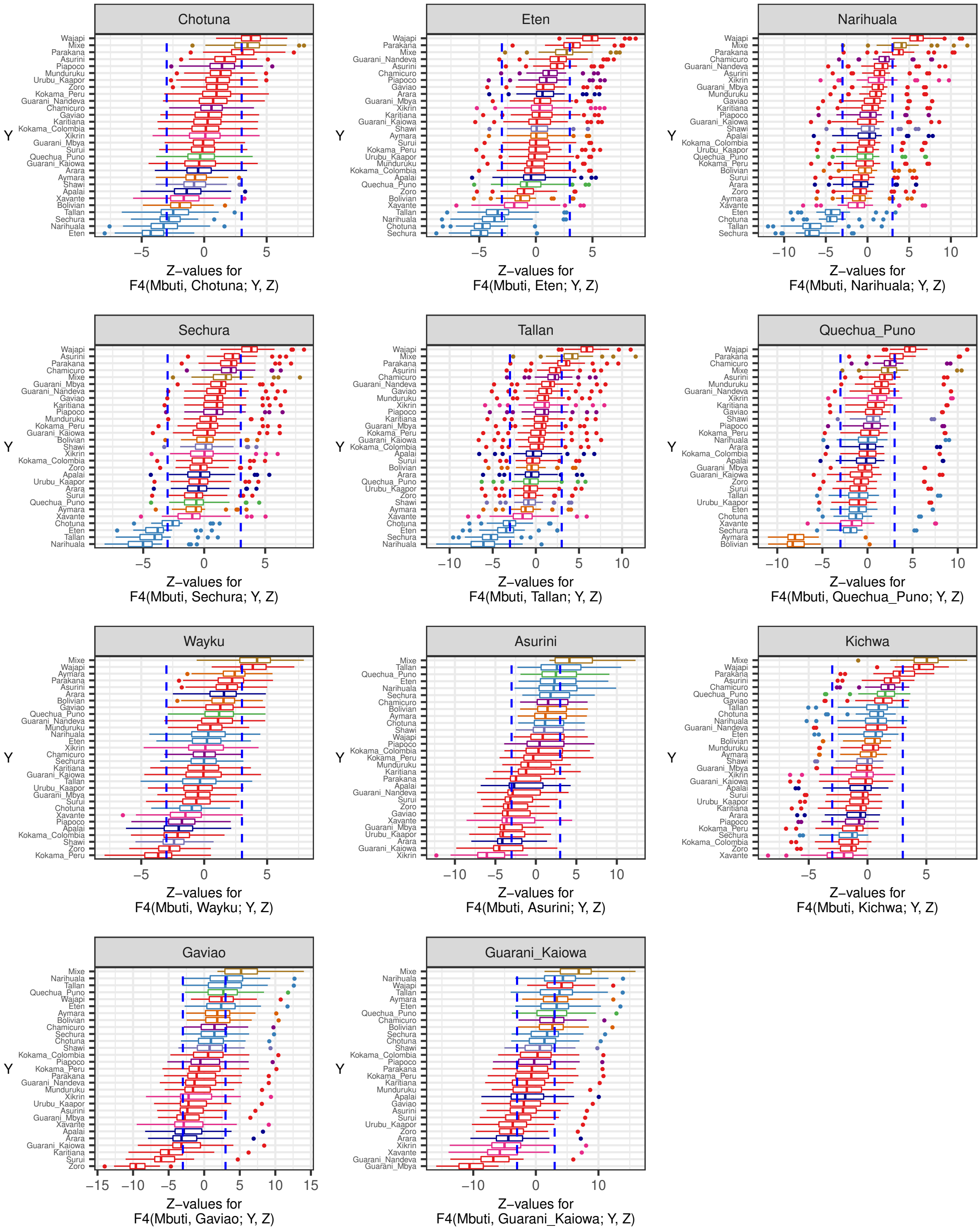

Figure S12 (continued)

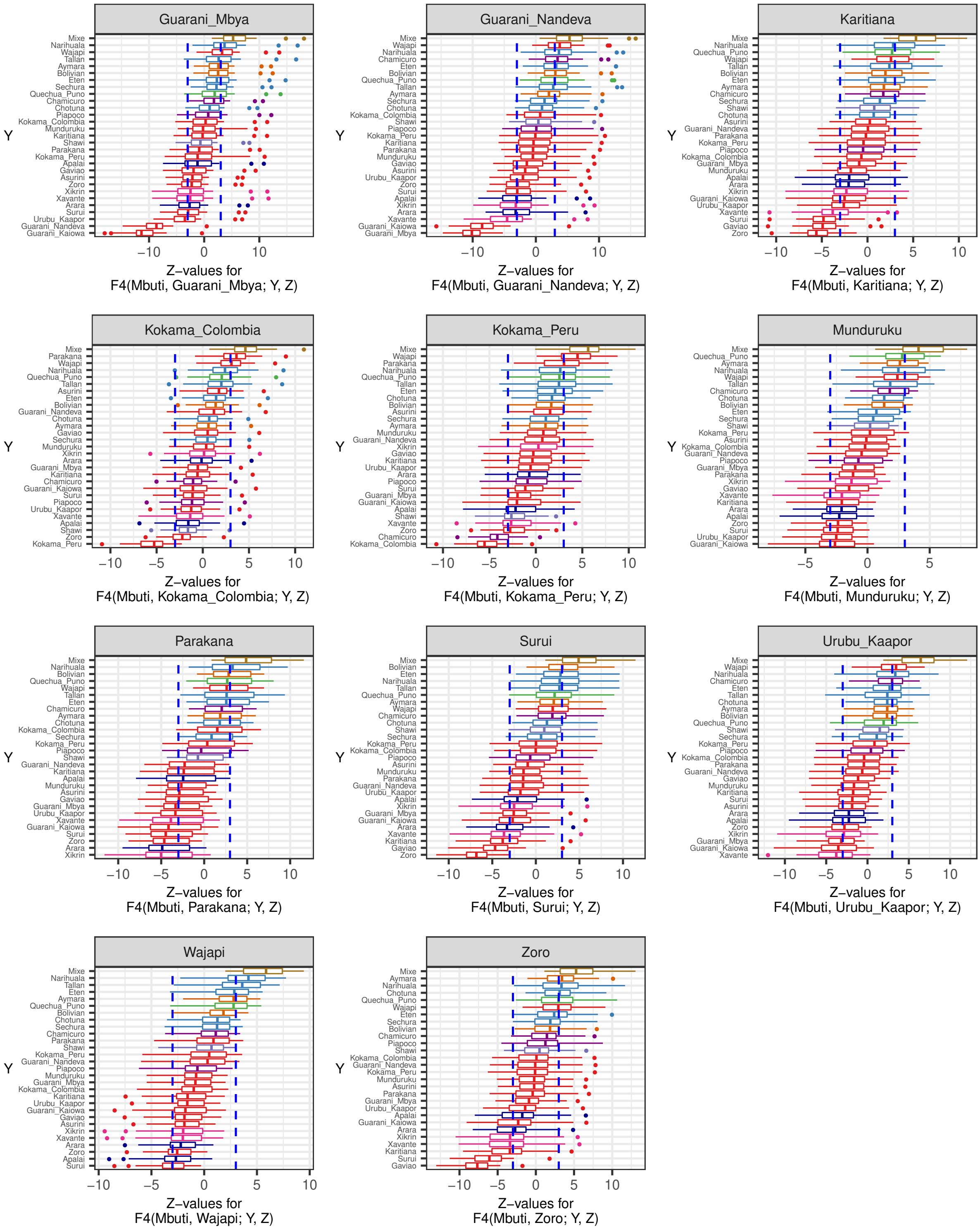

Figure S12 (continued)

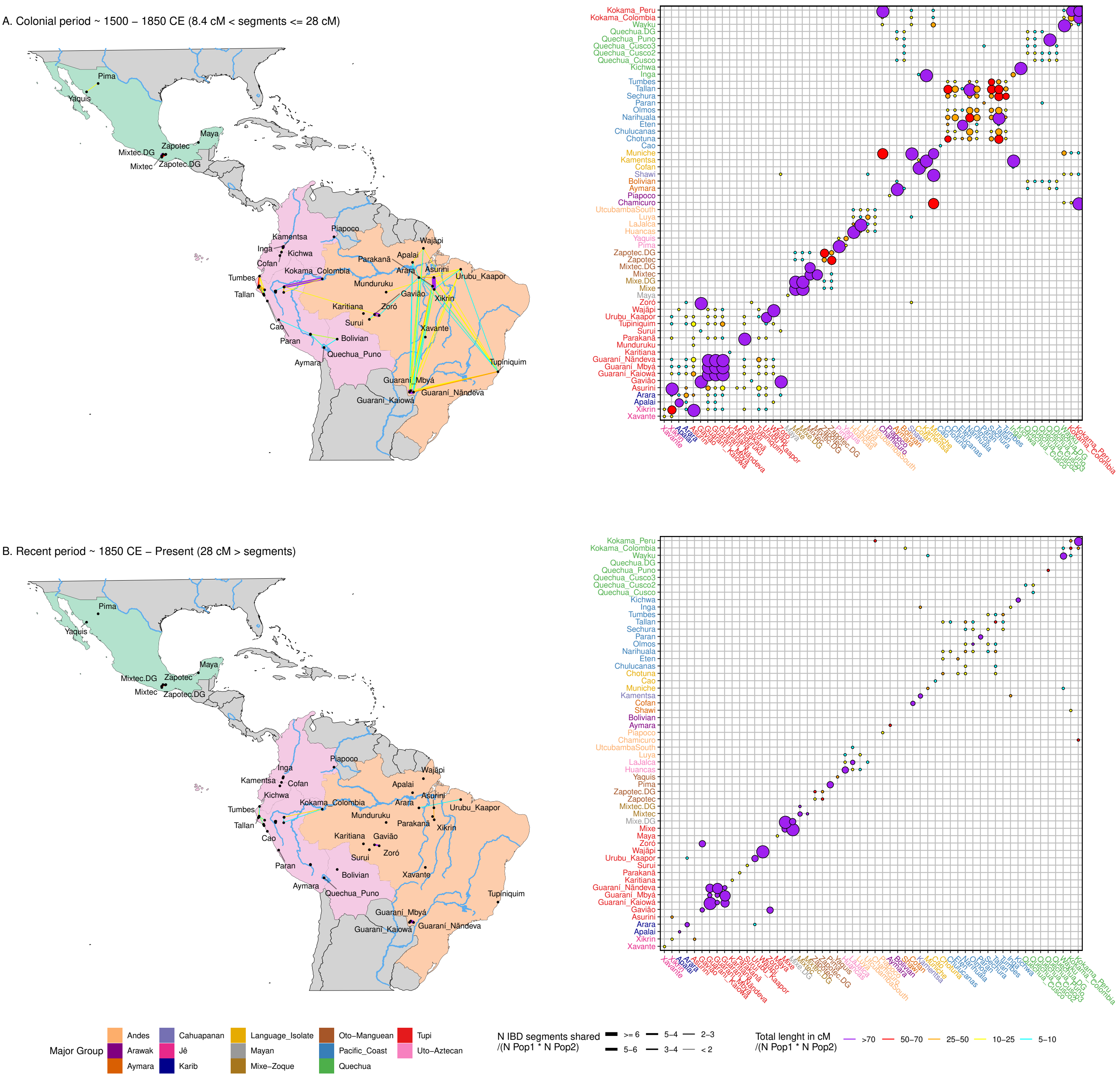

Figure S13 - Genetic connections among Native American groups. Here we present two networks and matrices created by estimating the average length of total IBD sharing and the average number of IBD segments, based on the same subsets of IBD genomic segments with length **(A)** between 9 and 22 cM and **(B)** with more than 22 cM. In this sense, the IBD genomic segments were identified based on the phased data subset of unrelated Native Americans, then they were filtered to select only those inferred to be in genomic regions of Native American local ancestry. We also removed segments shorter than 2 cM and pairwise connections with less than 5 cM shared on average were also not considered. Each map and matrix exhibits the average number of IBD segments (color) and the average length of IBD in cM (size), as indicated in the legends at the bottom. The three main continental regions are indicated by the colors used in each map. The complete set of IBD segments inferred are presented in Dataset S4.

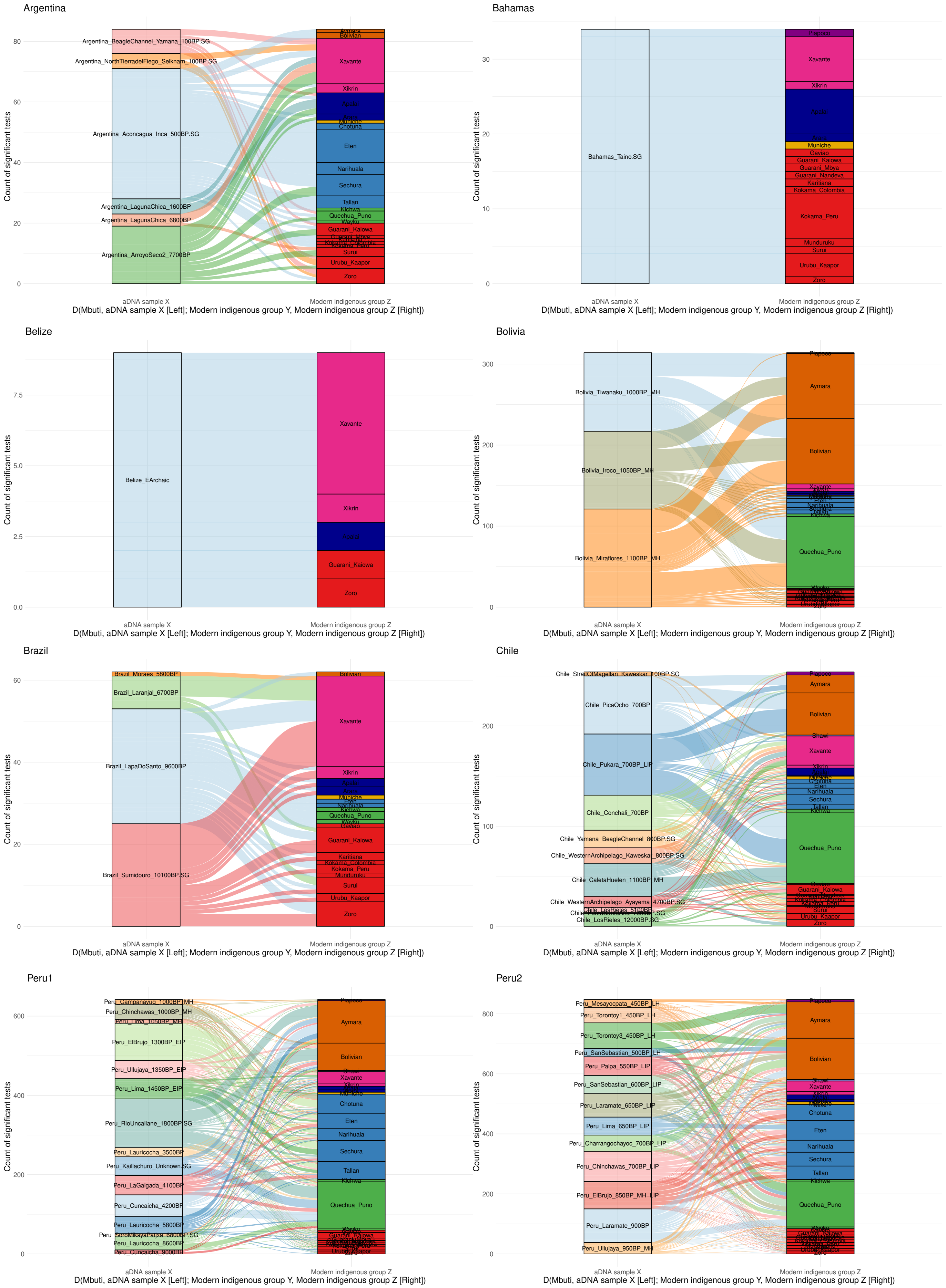

Figure S14 - Genetic affinities between present-day indigenous groups and ancient individuals (aDNA). To examine the patterns of allele sharing we estimated F_4(Mbuti, X; Y, Z) for every combination of X ancient individuals, and Y and Z present-day Native American groups. Ancient individuals from different countries are shown in separate panels, as indicated in their top left. Each panel shows the number of highly significant F_4 statistics (i.e. Z-value > 4) in the y-axis for each pair of X aDNA sample (left) and Z present-day indigenous

group (right), iterating over all Y groups. Metadata for the ancient samples used are included in Dataset S3 and the complete set of statistics is present in Dataset S6B.

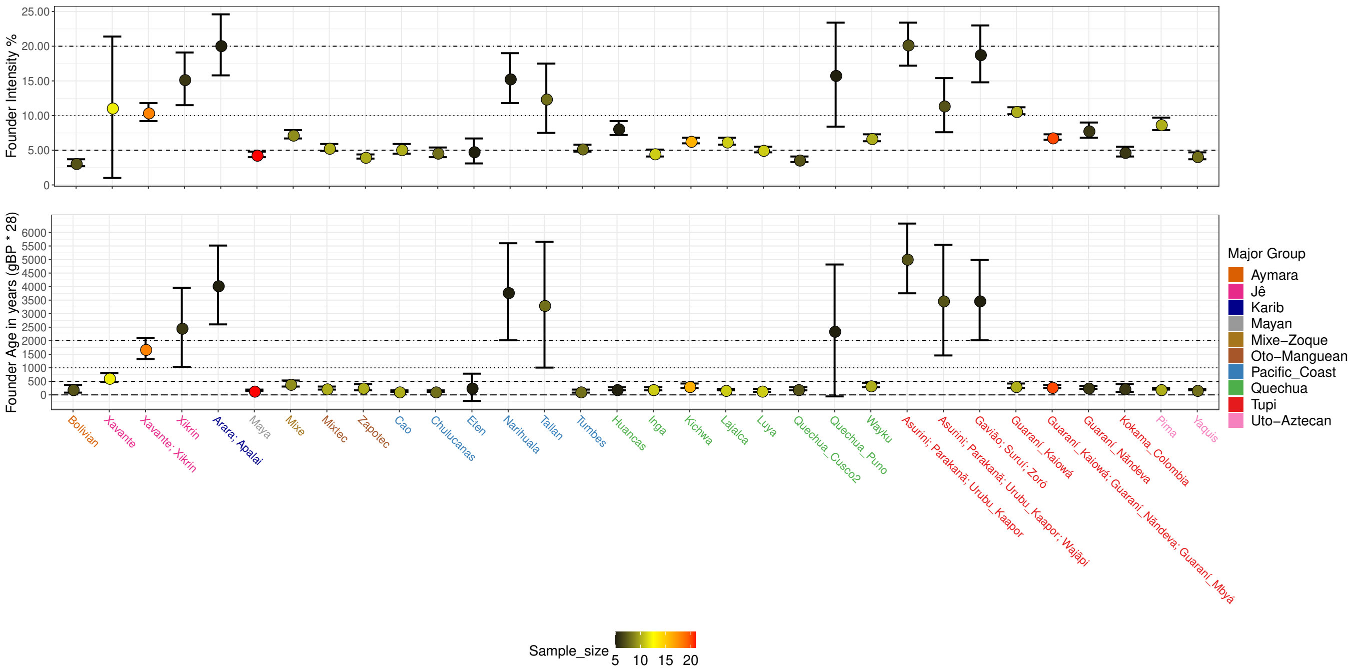

Figure S15 - Population bottlenecks in the history of Central and South American natives. The ASCEND method was applied to Native American groups with more than 5 unrelated samples and also to some clusters of groups (in order to reach the minimum sample size of 5). Here are presented the estimates of the complete set of clusters and groups **(A)** (the subset containing only the estimates for groups is presented in Figure 8). The top panels depict the FI and the bottom panels exhibit the mean estimate for the FA for each indigenous group or cluster of groups. For each group, the estimated FI and FA are shown, along with their associated 95% confidence interval. The sample size is color-coded on the points and the affiliations with major groups are indicated in the label IDs at the x-axis, both indicated in the legend. In the top panels, the y-axis indicates the FI percentage and in the bottom panel, the y-axis exhibits the estimated FA calculated as: ‘x’ generation before present (gBP) * 28 years per generation = ‘y’ years before present (BP).

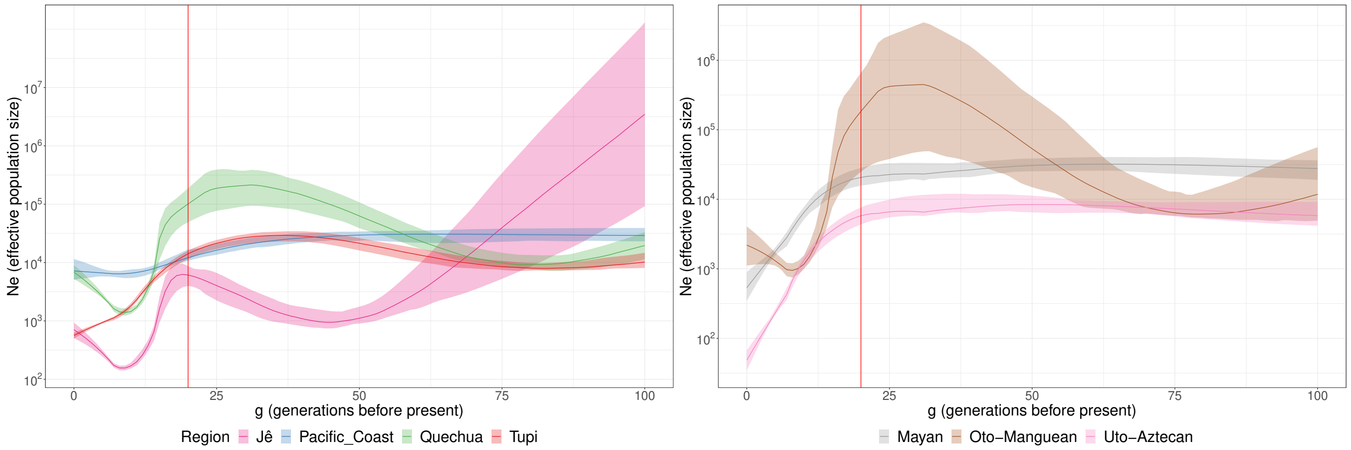

Figure S16 - Native American effective population size (N_e) histories. The IBD genomic segments were identified with the phased data subset of Native American groups, followed by a selection of the segments inferred to be in genomic regions of Native American ancestry. The complete set of IBD segments was separated into subsets of major groups (Table 1) from South America **(left)** and Mesoamerica **(right)**, and then each set was used to infer the N_e history of each specific major group. The ancestry-specific Ne values are coded in the y axis (log scale) and indicated by the line for each generation before the present (gBP) depicted in the x-axis. The shaded areas show a 95% bootstrap confidence interval for each major group. The vertical red line indicates 20 gBP (approximately 1500 C.E.) and therefore the time of the first contact with Europeans. Here we show the results of IBDNe using the parameter filtersampes = "true", alternatively the results produced with the parameter filtersampes = "false" are shown in Figure 8.

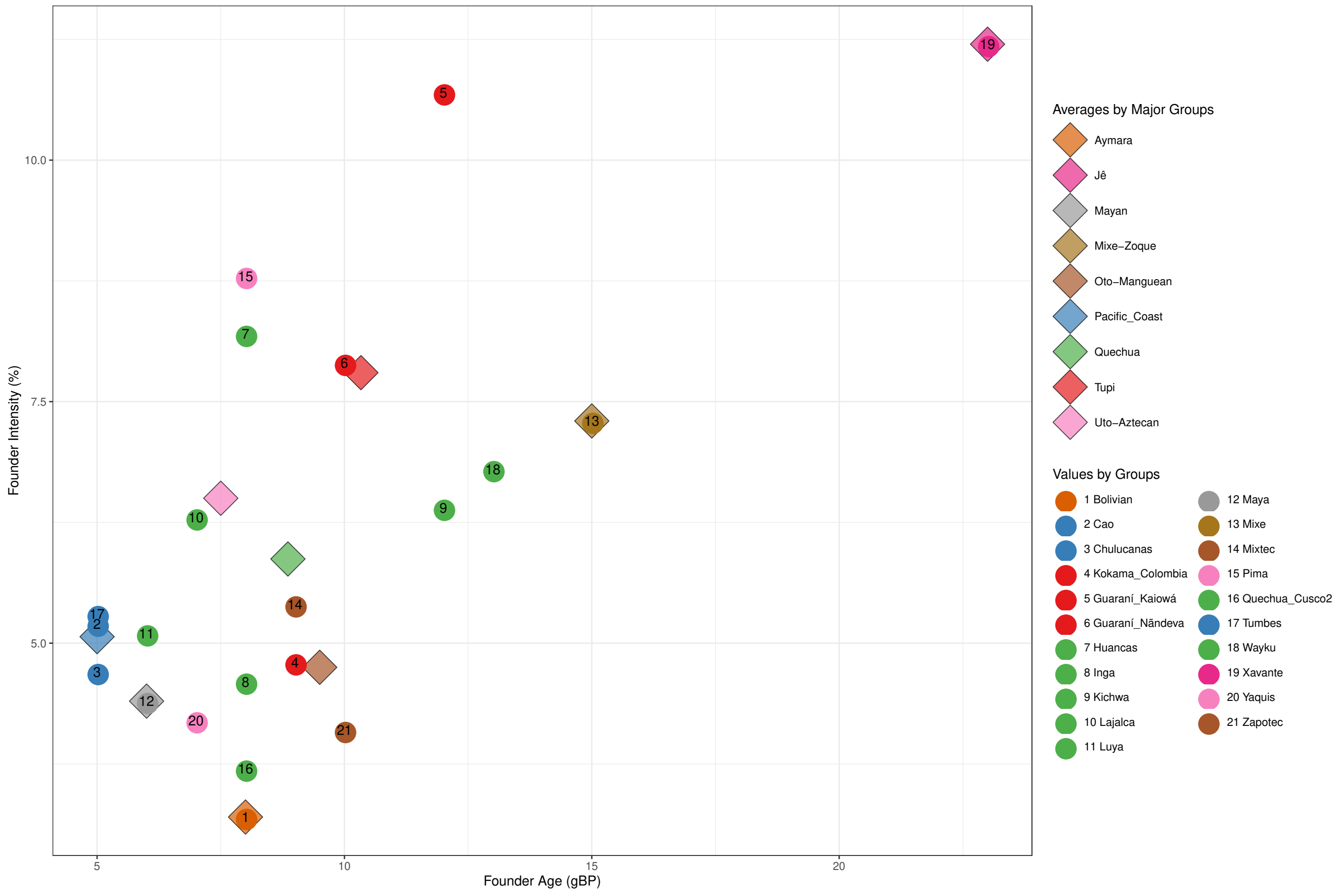

Figure S17 - Post-Contact population collapse founder intensity and founder age. (A) We applied the ASCEND (Tournebize et al. 2020) method to every Native American group with more than 5 unrelated samples, we also selected the groups with an estimated FA lower than 1000 BP as in Figure 6. The y-axis indicates the FI (in percentage) and the x-axis shows the mean estimate of the FA (in generations before present), values for each indigenous group are shown as circles and average values for each linguistic group are depicted as diamonds, both color-coded as in the legend.

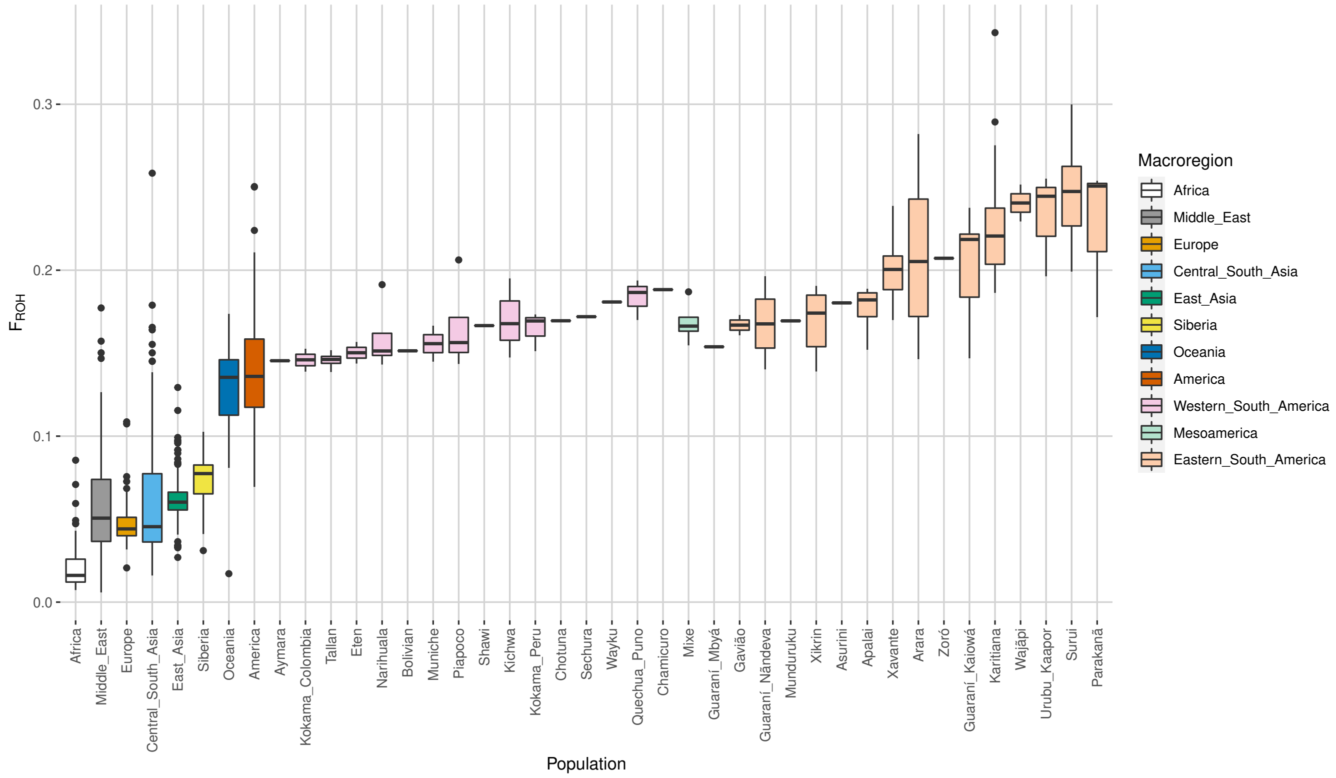

Figure S18 - Distribution of inbreeding coefficient per population. The distribution of F_{ROH} was obtained averaging the individual estimates from HGDP and SGDP databases (Africa, Middle East, Europe, Central South Asia, East Asia, Siberia, and America) and from each unadmixed Native American population independently.

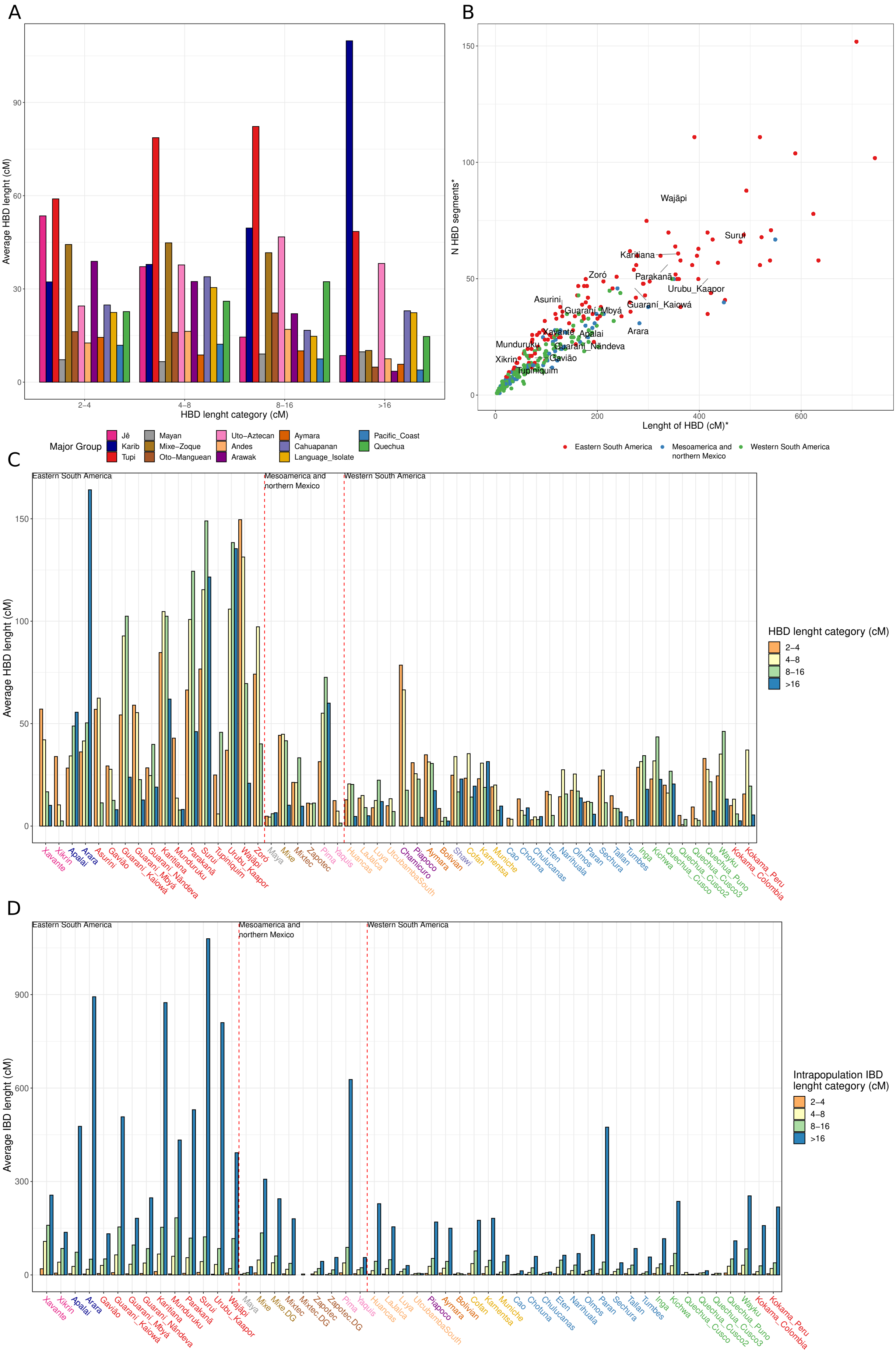
A
B
C
D
Average HBD lenght (cM)
HBD lenght category (cM)
N HBD segments*
Lenght of HBD (cM)*
Major Group
Jê
Karib
Tupi
Mayan
Mixe–Zoque
Oto–Manguean
Uto–Aztecan
Andes
Arawak
Aymara
Cahuapanan
Language_Isolate
Pacific_Coast
Quechua
Eastern South America
Mesoamerica and northern Mexico
Western South America
Average IBD lenght (cM)
Intrapopulation IBD lenght category (cM)

Figure S19 - Patterns of intrapopulation sharing of identity-by-descent (IBD) and homozygosity-by-descent (HBD) segments. The IBD and HBD genomic segments were identified with Refined IBD (Browning et al. 2018), based on the phased subset of Native American populations. Only IBD segments located in regions of local Native American ancestry as inferred with RFMix (Maples et al. 2013) were used. **A)** The average HBD total length (y-axis) was estimated for each major group (Table 1; color-coded) and binned into 5 segment length categories (x-axis). **B)** Number of HBD segments (y-axis) plotted against the total length of HBD in cM (x-axis). *Absolute values for all individuals and average values for eastern South American groups are represented by filled circles and labels, respectively. Color code indicates whether the group or individual is from Mesoamerica and northern Mexico, eastern South America, or western South America. **C)** The average HBD total length (y-axis) was estimated for each group (x-axis) and binned into 5 segment length categories (color-coded). **D)** The average IBD length shared between individuals from the same groups (y-axis) was estimated for each group (x-axis; some groups are absent, as they have only 1 individual or none intra-population shared IBD) and binned into 4 segment length categories (color-coded; the category 1–2 cM is absent). In **C** and **D**, populations are sorted by geographic location, from left to right: eastern South America (Xavante to Wajãpi), Mesoamerica and northern Mexico (Maya to Yaquis), and western South America (Piapoco to Kokama Peru), as indicated by the red dashed lines and labels at the top of the panel.

Supplementary Data Files

Dataset S1 - Metadata for test samples. This dataset presents metadata for each present-day Native American individual used in our analyses, the information includes for example original group name (as used in the data source study), group name (as used in this study), individual ID, major ethnolinguistic group affiliation, country of origin, data source study, data source method (e.g. Axiom Human Origins array or Shotgun sequencing), geographic coordinates, inclusion on the maximum unrelated dataset (True or False) and presence of non-Native American admixture (True or False). This dataset also presents estimates produced by an unsupervised ADMIXTURE (Alexander et al. 2009) analysis on the subset of Native Americans with K = 3. These estimates are the same presented in Figure S1. Finally, the colors used to represent each individual throughout the study is also included.

Dataset S2 - Metadata for reference samples. Here we present metadata for each individual from a reference population used in our analyses, the information includes group name, individual ID, country of origin, data source study, macro-region of origin, continent of origin, data source method (e.g. Axiom Human Origins array or Shotgun sequencing).

Dataset S3 - Metadata for ancient samples (aDNA). This dataset contains metadata for each ancient individual sample used in our analyses, as obtained from 1240K+HO (v42.4; https://reichdata.hms.harvard.edu/pub/datasets/amh_repo/curated_releases/index_v42.4.html) curated dataset, including information on individual IDs, study publication, representative contact, date mean in BP, date CIs, group label, locality, country, geographic coordinates, data source method (e.g. 1240K or Shotgun sequencing), coverage, library type, and quality assessment.

Dataset S4. Inferred IBD segments of Native American ancestry. This dataset presents the complete set of IBD segments inferred from genomic regions of Native American local ancestry based on the subset of unrelated present-day Native Americans and includes information about group identification, individual identification, haplotype index, chromosome, starting and ending genomic positions, and length in cM for each IBD segment.

Dataset S5. Estimates of outgroup F_3(Y, Z; Mbuti). This dataset includes the F_3-statistics for every combination of Y and Z indigenous groups **(A)** or individuals **(B)**, in the unadmixed and unrelated subset of present-day Native Americans, as well as the F_3-statistics for every pair of Y and Z present-day and/or ancient individuals **(C)**. The datasets include information on F_3-statistic, standard error (SE), Z-value, and the total number of shared SNPs across the tested populations.

Dataset S6. Estimates of F_4(Mbuti, X; Y, Z). This dataset presents **(A)** the F_4-statistics F_4(Mbuti, X; Y, Z) for every combination of X, Y, and Z present-day American indigenous groups; and also contains **(B)** the F_4-statistics F_4(Mbuti, X; Y, Z) for every combination of X ancient individuals, with pairs of Y and Z present-day American indigenous groups. Present-day samples include only those present in the unadmixed and unrelated subset. The datasets present information on F_4-statistic, Z-Value, number of ABBA and BABA positions, and the total number of shared SNPs across the tested populations.

References

Alexander DH, Novembre J, Lange K. 2009. Fast model-based estimation of ancestry in unrelated individuals. *Genome Res.* 19:1655–1664.

Behr AA, Liu KZ, Liu-Fang G, Nakka P, Ramachandran S. 2016. pong: fast analysis and visualization of latent clusters in population genetic data. *Bioinformatics* 32:2817–2823.

Browning SR, Browning BL, Daviglus ML, Durazo-Arvizu RA, Schneiderman N, Kaplan RC, Laurie CC. 2018. Ancestry-specific recent effective population size in the Americas. *PLoS Genet.* 14:e1007385.

Excoffier L, Smouse PE, Quattro JM. 1992. Analysis of molecular variance inferred from metric distances among DNA haplotypes: application to human mitochondrial DNA restriction data. *Genetics* 131:479–491.

Kamvar ZN, Tabima JF, Grünwald NJ. 2014. Poppr: an R package for genetic analysis of populations with clonal, partially clonal, and/or sexual reproduction. *PeerJ* 2:e281.

Lazaridis I, Nadel D, Rollefson G, Merrett DC, Rohland N, Mallick S, Fernandes D, Novak M, Gamarra B, Sirak K, et al. 2016. Genomic insights into the origin of farming in the ancient Near East. *Nature* 536:419–424.

Lazaridis I, Patterson N, Mittnik A, Renaud G, Mallick S, Kirsanow K, Sudmant PH, Schraiber JG, Castellano S, Lipson M, et al. 2014. Ancient human genomes suggest three ancestral populations for present-day Europeans. *Nature* 513:409–413.

Maples BK, Gravel S, Kenny EE, Bustamante CD. 2013. RFMix: a discriminative modeling approach for rapid and robust local-ancestry inference. *Am. J. Hum. Genet.* 93:278–288.

Pickrell JK, Pritchard JK. 2012. Inference of population splits and mixtures from genome-wide allele frequency data. *PLoS Genet.* 8:e1002967.

Staples J, Nickerson DA, Below JE. 2013. Utilizing graph theory to select the largest set of unrelated individuals for genetic analysis. *Genet. Epidemiol.* 37:136–141.

Tournebize R, Chu G, Moorjani P. 2020. Reconstructing the history of founder events using genome-wide patterns of allele sharing across individuals. *bioRxiv* [Internet].
